# Endocrine and molecular milieus of ovarian follicles are diversely affected by human chorionic gonadotropin and gonadotropin-releasing hormone in prepubertal and mature gilts

**DOI:** 10.1038/s41598-021-91434-6

**Published:** 2021-06-29

**Authors:** Adam J. Ziecik, Jan Klos, Katarzyna Gromadzka-Hliwa, Mariola A. Dietrich, Mariola Slowinska, Pawel Likszo, Katarzyna Knapczyk-Stwora, Zdzislaw Gajewski, Monika M. Kaczmarek

**Affiliations:** 1 Department of Hormonal Action Mechanisms, Institute of Animal Reproduction and Food Research, Polish Academy of Sciences, Tuwima 10 Str., 10-747 Olsztyn, Poland; 2 Department of Gamete and Embryo Biology, Institute of Animal Reproduction and Food Research, Polish Academy of Sciences, Olsztyn, Poland; 3 Department of Endocrinology, Institute of Zoology and Biomedical Research, Jagiellonian University, Krakow, Poland; 4 Center for Translational Medicine, Warsaw University of Life Sciences, Warsaw, Poland; 5 Molecular Biology Laboratory, Institute of Animal Reproduction and Food Research, Polish Academy of Sciences, Olsztyn, Poland

**Keywords:** Physiology, Reproductive biology

## Abstract

Different strategies are used to meet optimal reproductive performance or manage reproductive health. Although exogenous human chorionic gonadotropin (hCG) and gonadotropin-releasing hormone (GnRH) agonists (A) are commonly used to trigger ovulation in estrous cycle synchronization, little is known about their effect on the ovarian follicle. Here, we explored whether hCG- and GnRH-A-induced native luteinizing hormone (LH) can affect the endocrine and molecular milieus of ovarian preovulatory follicles in pigs at different stages of sexual development. We collected ovaries 30 h after hCG/GnRH-A administration from altrenogest and pregnant mare serum gonadotropin (eCG)-primed prepubertal and sexually mature gilts. Several endocrine and molecular alternations were indicated, including broad hormonal trigger-induced changes in follicular fluid steroid hormones and prostaglandin levels. However, sexual maturity affected only estradiol levels. Trigger- and/or maturity-dependent changes in the abundance of hormone receptors (FSHR and LHCGR) and proteins associated with lipid metabolism and steroidogenesis (e.g., STAR, HSD3B1, and CYP11A1), prostaglandin synthesis (PTGS2 and PTGFS), extracellular matrix remodeling (MMP1 and TIMP1), protein folding (HSPs), molecular transport (TF), and cell function and survival (e.g., VIM) were observed. These data revealed different endocrine properties of exogenous and endogenous gonadotropins, with a potent progestational/androgenic role of hCG and estrogenic/pro-developmental function of LH.

## Introduction

A natural model to gauge the estrous cycle includes weaned sows or postpubertal gilts monitored for estrous behavior^42^^,^^39^. Unfortunately, even accurate estrus detection will not compensate for the variability in the interval between the onset of estrus and the actual time of ovulation. Hormonal treatments have been used in different protocols to control the reproductive functions of sows and gilts^16^, allowing overcoming this variability and synchronizing ovulation. For example, postpubertal (sexually mature) gilts are often synchronized with the progesterone receptor agonist altrenogest^21,29^, which is administered to calm down the hypothalamic–pituitary–ovarian axis and inhibit follicular development. However, proper identification of mature gilts for altrenogest treatment can be tricky, as Tummaruk et al.^58^ showed that only 33% of gilts ovulated in the first estrus, 21% ovulated before showing the first behavioral estrus and 45% did not ovulated during the first estrus. Thus, special attention should be paid to confirm ovulation, as too early hormonal treatment may lead to development of follicular cysts in prepubertal gilts^66^. Our recent studies have indicated that in addition to its known effects on ovarian folliculogenesis and development of antral follicles, altrenogest causes multiple changes in the endocrine milieu of follicles in prepubertal and mature gilts^68^.

Routine synchronization of the estrous cycle in pigs and other domestic animal species (except the pituitary–ovary quiescence period) comprises two distinct phases: stimulation of follicular growth and induction of ovulation with exogenous gonadotropins. Both prepubertal and mature gilts are usually challenged with pregnant mare gonadotropin (eCG) to stimulate follicular growth, followed by administration of exogenous human chorionic gonadotropin (hCG)^3^ or gonadotropin releasing hormone (GnRH) agonist (A) to induce the release of endogenous luteinizing hormone (LH)^43^. Although hCG acts directly through ovarian receptors, GnRH-A stimulates pituitary LH release, which then reaches the ovary to aid the ovulation process.

The abovementioned models provide a good approximation of the native state, allowing to control some variability among animals, but they do not exhibit steroidogenesis identical to naturally cycling animals^39^. Differences in follicular steroidogenesis and oocyte maturation in naturally cyclic and eCG/hCG-treated prepubertal gilts have been reported^63^. Furthermore, treatment with exogenous gonadotropins combined with altrenogest leads to multiple ovarian follicular cysts in sexually immature gilts^66^. Still, hCG and GnRH-A are routinely used to synchronize ovulation in gilts and sows^19^.

Both LH and hCG activate the same receptor, LHCGR, and similarly induce testosterone synthesis in Leydig cells in vitro^47,51^. However, in goat^28^ and human^10^ ovarian granulosa cells, different responses in duration, strength, and timing have been observed. hCG showed greater affinity to LHCGR, with a five-fold greater potency to increase cyclic adenosine monophosphate (cAMP) production, whereas LH preferentially activated extracellular signal-regulated kinases (ERK1/2 and protein kinase B)^10^. It seems likely that hCG has a stronger steroidogenic signal, but LH exerts stronger antiapoptotic action. In addition, hCG has a considerably longer half-life than LH (28 h vs. 20 min; respectively)^1^.

In porcine granulosa and theca cells, recombinant (r)-LH and r-hCG had similar effects in vitro, mirrored in identical cAMP generation dynamics. However, altrenogest treatment decreased hCG-stimulated cAMP production in the theca layers of prepubertal but not mature gilts^68^. Thus, we hypothesized that hCG can initiate earlier androgenization of 17-OH-pregnenolone and increase progesterone production (especially in the follicles of prepubertal gilts), whereas GnRH administration, causing LH release, can ensure better (estrogenic) endocrine milieu for the onset of undisturbed ovulation. Moreover, the much longer half-life of hCG than LH could cause alternations and stigmatization of preovulatory follicles toward cyst development. Thus, in prepubertal gilts, who do not have a fully developed hypothalamic–pituitary–ovarian axis, the response to GnRH-A and hCG is likely different from that in mature gilts primed with a progestagen.

Since hCG and LH have biased agonism^48^ at LHCGR, do not activate the same intracellular signaling pathways upon receptor binding^10^, and lead to different proliferative and antiapoptotic responses^11^, we hypothesized that such diverse responses to both gonadotropins should also affect the milieu of preovulatory follicles. To test this hypothesis, we challenged prepubertal and mature gilts with hCG or GnRH-A, evaluated ovarian follicle morphology and assessed the endocrine and molecular milieus of preovulatory follicles. This study is a continuation of our previous report concerning phenotypic variations in an ovarian response to pharmacological management of the reproductive cycle in pigs. Understanding how ovaries of prepubertal and sexually mature gilts respond to exogenous hCG and native LH may guide strategies for the reproductive management of gilts and sows. Our data may also be relevant to human reproductive medicine.

## Materials and methods

### Selection of animals and experimental group recruitment

The experiment was performed in accordance with the national and EU guidelines for agricultural animal care (EU Directive 2010/63/UE) and approved by the Local Animal Ethics Committee (University of Warmia and Mazury, Olsztyn, Poland; permission number: 38/2020).

Crossbred gilts at 165 days of age were contacted with mature boar every day for 14 days and then at approximately 180 days of age were used in two trials to create experimental groups. The detailed procedures are described in our recent paper^68^. Briefly, gilts considered as being in the first natural estrus formed a set of future sexually mature (M) gilts, which were recruited at 185–195 days of age. A set of gilts without estrus symptoms at 180 days of age was designed to form prepubertal (P) groups. The sexual maturity was defined based on a fully expressed first estrus and occurrence of ovulations, confirmed by the presence of *corpora albicantia* after ovariectomy.

Gilts of both M (n = 10) and P (n = 12) sets were fed 20 mg of altrenogest (Suifertil, Medica, Poland) daily administered (5 mL) orally with the Suifertil pump for 18 consecutive days. The day after the last treatment (day 19), all gilts were treated i.m. with 750 IU eCG (500 IU- j.m., Syncrostim, Ceva Santé Animale, Libourne, France) and 48 h later (day 21), each M and P group was divided into two subgroups (n = 5–6) and challenged with hCG (500 IU Chorulon, Intervet International Boxmeer, Nederland) or GnRH-A (50 µg i.m. Depherelin, Veyx-Pharma GmbH, Schwarzenborn, Germany). In consequence, two prepubertal hCG (n = 6), GnRH-A (n = 6), and two mature hCG (n = 5) and GnRH-A (n = 5) challenged groups were formed. Prepubertal and mature groups were ovariectomized 30 h after hCG or GnRH-1 administration at 200–206 days of age and 128–135 kg body weight.

This protocol allowed certain experimental goals to be achieved. Specifically, hCG and GnRH-A challenge 48 h after eCG (but not 72 h) was performed according to our original protocol^66^ to avoid premature rupture of preovulatory follicles before the administration of two tested ovulation stimuli. In all experimental gilts, ovaries were collected before ovulation during ovariectomy preformed 30 h after hCG or GnRH-A injection.

### Sample collection

Both ovaries were collected from all gilts (P, M) during ovariectomy and placed in ice-cold phosphate-buffered saline (137 mM NaCl, 27 mM KCl, 10 mM Na_2_HPO_4_, and 2 mM KH_2_PO_4_; pH 7.4), containing 100 IU of penicillin (Sigma-Aldrich, Saint Louis, MO, USA) and 100 μg/mL of streptomycin (Sigma-Aldrich). Ovaries were weighed, placed against a ruler, and photographed from different sides to count preovulatory follicles (< 6 mm, 6–8 mm, and > 8 mm). Next, follicular fluid was collected by aspiration (4–5 preovulatory follicles per animal) from both ovaries and pooled, centrifuged at 1550 × g for 10 min at 4 °C to remove cell debris, and frozen at − 20 °C until assayed for hormone concentrations. Follicular walls were separated by cutting out and peeling off the same follicle. After collection, the follicular walls were placed in a clean tube, snap-frozen in liquid nitrogen, and kept at − 80 °C for further analysis. One follicle from each ovary was assigned for proteomic analysis.

In addition, ovaries with preovulatory follicles from prepubertal and mature gilts were collected at a local slaughterhouse and fixed in Bouin solution (Sigma-Aldrich) to be used for further immunohistochemical staining.

### Prostaglandin and steroid hormone assays

Steroid hormone concentration in follicular fluid was determined using radioimmunoassay (RIA) kits: A4-RIA-CT for androstenedione (A_4_), E2-RIA-CT for estradiol-17-beta (E_2_), T-RIA-CT for testosterone (T), and PROG-RIA-CT for progesterone (P_4_; all from DIASource, Louvain-le-Neuve, Belgium), according to the manufacturer’s instructions. Assay sensitivity was 0.03 ng/mL for A_4_, 2.7 pg/mL for E_2_, 0.5 ng/mL for T and 0.05 ng/mL for P_4_, and intra-assay coefficients of variation were 5.9%, 10.4%, 6.5%, and 8.3%, respectively.

Prostaglandin (PG) E_2_ and 13,14-dihydro-15-keto PGF_2α_ (PGFM) concentration in follicular fluid was determined using the conventional EIA method according to Blitek et al.^7^. Anti-PGE_2_ antibodies and anti-PGFM (donated by Dr. W. Silvia, University of Kentucky, Lexington, KY, USA, Supplementary Table 1) developed in rabbits were used to determine PGE_2_ and PGFM in the follicular fluid. The sensitivity of the assay was 0.19 ng/mL for PGE_2_ and 25 ng/mL for PGFM. The intra-assay coefficients of variation were 9.4% for PGE_2_ and 12.3% for PGFM.

### Immunohistochemistry

Immunohistochemical analysis was performed for antral preovulatory follicles collected from prepubertal and mature gilts at the slaughterhouse to localize transferrin (TF) and vimentin (VIM). Preovulatory follicle walls were fixed, sectioned, and mounted for immunohistochemistry, as previously described by Ziecik et al.^68^. Subsequently, sections were incubated in 0.3% (v/v) hydrogen peroxide in Tris-buffered saline (TBS, 0.1 M Tris and 150 mM NaCl; pH 7.4) for 30 min at room temperature to block endogenous peroxidase activity and treated with 5% (v/v) normal goat serum (for TF) or 5% (v/v) normal horse serum (for VIM) at room temperature for 30 min to block nonspecific binding sites. For immunolabeling, sections were incubated overnight at 4 °C with the rabbit anti-transferrin polyclonal antibody or the mouse anti-vimentin monoclonal antibody (Supplementary Table 1), rinsed in TBS with 0.1% (v/v) Tween 20 (TBS-T), and incubated for 1.5 h at room temperature with goat anti-rabbit or horse anti-mouse biotinylated secondary antibody (Supplementary Table 1). Next, incubation with avidin–biotin-peroxidase complex (StreptABComplex-HRP, Vector Laboratories, Burlingame, CA) for 40 min was performed. Immune complexes were visualized using 3,3’-diaminobenzidine (Sigma-Aldrich) as a chromogen. For the negative control reaction, sections were incubated with nonimmune rabbit or mouse IgG instead of primary antibodies and processed as above. In a final step, slides were dehydrated, fixed in xylene, and mounted using DPX (Sigma-Aldrich) and coverslips. Sections were photographed under a Nikon Eclipse Ni-U light microscope using a Nikon Digital DS-Fi1-U3 camera (Nikon, Tokyo, Japan) with corresponding software.

### Protein extraction

For Western blotting, walls of preovulatory follicles (granulosa and theca layers) of P and M gilts were homogenized by sonication (Sonopuls, Bandelin Electronic GmbH & Co. KG, Berlin, Germany) on ice in lysis buffer (50 mM Tris–HCl, pH 7.4; 150 mM NaCl; 1% Triton X-100 (v/v); 0.02% sodium azide and 1 mM/L EDTA) containing 100 mM protease inhibitor cocktail (Sigma-Aldrich). The homogenates were then centrifuged at 800 × g for 10 min at 4 °C and stored at − 80 °C until analysis. Protein concentration was determined using the Bradford method^9^.

For two-dimensional difference gel electrophoresis (2D-DIGE), walls of preovulatory follicles (granulosa and theca layers) were sonicated using VC-13 PB (Sonics, Newtown, CT, USA) in lysis buffer containing 7 M urea, 2 M thiourea, 2% CHAPS, 2% immobilized pH gradient (IPG) buffer 3–10 NL, 100 mM dithiothreitol, 1% Triton X-100, and 0.5% protease inhibitor cocktail (Sigma-Aldrich). Next, the samples were kept on ice for 60 min and centrifuged at 12,000 × g for 20 min at 4 °C. After centrifugation, protein samples were precipitated using a Clean-up Kit (GE Healthcare, Uppsala, Sweden) according to the manufacturer’s protocol. The precipitate was dissolved in labeling buffer (7 M urea; 2 M thiourea; 4% w/v CHAPS; 30 mM Tris, pH 8.0). The protein concentration before and after precipitation was determined with the Bradford method^9^ by using a Coomassie Plus Kit (Thermo Fisher Scientific, Waltham, MA, USA) with bovine serum albumin as a standard.

### 2D-DIGE

Samples were labeled with CyDye DIGE Fluor minimal dye (GE Healthcare) reconstituted in fresh 99.8% anhydrous dimethylformamide at 50 μg protein to 400 pmol Fluor dye. The labeling reaction was performed on ice in the dark for 30 min. Samples from ovarian follicles of prepubertal and mature hCG- or GnRH-A-treated gilts were labeled with Cy3 or Cy5 according to the scheme provided by the manufacturer (Supplementary Table 2). Two extra samples (technical replicates; mature hCG- [n = 1] and GnRH-A-treated [n = 1]) were added to complete labelling scheme suggested by the manufacturer. A dye-swap (Cy3/Cy5) was performed between samples to exclude dye bias. The internal standard was generated by combining equal amounts of each sample within the experiment and was labeled with Cy2. An equal amount of Cy2-labeled pooled standard was loaded on each gel for normalization and to correct for gel-to-gel variability. After the labeling reaction, samples were mixed (Cy2, Cy3, and Cy5) according to the scheme presented in Supplementary Table 2. Next, each sample mixture was added to rehydration buffer (7 M urea, 2 M thiourea, 2% CHAPS, 10 mM DTT, 2% v/v IPG buffer pH 3–10 and 0.002% bromophenol blue) to reach a final volume of 450 µL. The protein samples were loaded on 24 cm Immobiline DryStrips, pH 3 to 10 nonlinear pH gradient (GE Healthcare) and rehydrated for 18 h (passive rehydration). Isoelectric focusing was performed with an IPGphor isoelectric focusing unit (GE Healthcare), and sodium dodecyl sulfate–polyacrylamide gel electrophoresis (SDS-PAGE) was run using the ETTAN Dalt six electrophoresis unit (GE Healthcare) as described by Ciereszko et al.^14^.

### Image and data analysis

Four groups of proteins (prepubertal GnRH-A, prepubertal hCG, mature GnRH-A, and mature hCG) of preovulatory follicle walls (granulosa and theca layers) were resolved using 2D-DIGE. The obtained gels were scanned with a Typhoon 9500 FLA scanner (GE Healthcare) using the parameters suggested by the manufacturer’s instructions. The scanned images were analyzed with DeCyder Differential Analysis software version 5.02 (GE Healthcare) to identify differences in fluorescence intensities of the spots. During spot detection, the estimated number of spots was set at 10,000 and volume < 30,000. Protein spots with a *P* < 0.05 by one-way analysis of variance (ANOVA), which indicated an increase or decrease in relative intensity (in-gel ratios greater than 1.15), were considered differentially abundant proteins. Only spots that were successfully matched on > 80% of the gel images were considered for further analysis. To properly select and identify the spots, gels were stained using Coomassie Brilliant Blue G250 after 2D-DIGE.

### Protein identification by mass spectrometry

Spots of interest were cut from the 2D-DIGE gel and prepared for identification using a MALDI-TOF tandem mass spectrometer (Autoflex Speed, Bruker Daltonics) as previously described by^14^. The MS peptide mass fingerprint and fragment mass spectra (MS/MS) from each spot were combined and used to search against the National Centre for Biotechnology Information Sus scrofa database (searched on December 4, 2019) using the Mascot Server (Matrix Science, London, UK) with the following settings: cleavage enzyme, trypsin,max missed cleavages, 2; fragment ion mass tolerance, 0.5 Da; parent ion mass tolerance, 100 ppm; alkylation of cysteine by carbamidomethylation as a fixed modification; and oxidation of methionine as a variable modification. The search results were filtered with a significant threshold of *P* < 0.05 and a Mascot ion score cutoff of ≥ 30 for at least two peptides.

### In silico functional analysis

Core analysis of proteins was implemented by Ingenuity® Pathways Analysis (IPA, Ingenuity Systems, ww.ingenuity.com), where proteins are analyzed using the biological function and predicted upstream regulators. Differentially expressed protein identifiers were defined as value parameters for analysis, and the relationship between protein expression was identified. IPA uses a network generation algorithm to segment the network map between molecules into multiple networks and assign scores for each network. The right-tailed Fisher’s exact test, using a threshold of *P* < 0.05 after application of the Benjamin–Hochberg method for multiple testing correction and z-score (for proteins with significantly altered abundances) were used as two statistical measures for identifying significant biofunctions and upstream regulators.

### Western blot

Total protein lysates from follicular walls were dissolved in SDS gel-loading buffer (250 mM/L Tris–HCl, pH 6.8; 10% β-mercaptoethanol; 125 mM SDS; 40% glycerol; and 0.578 mM bromophenol blue), denatured at 95 °C for 4 min, and separated on a TGX Stain-Free 10% gel (Bio-Rad, Hercules, CA, USA) at 48 mA for 1.5 h. Protein samples for MMP1 were separated on 10% SDS-PAGE. Before the transfer of protein onto the polyvinylidene difluoride membrane (Sigma-Aldrich), the TGX Stain-Free gels were activated to obtain the total content of loaded protein, according to the manufacturer’s instructions. Blotted membranes were washed in TBS-T and blocked in 5% nonfat dried milk in TBS-T for 1.5 h at room temperature. Next, membranes were immunoblotted overnight at 4 °C with polyclonal rabbit or mouse antibodies: anti-STAR, anti-HSD3B1, anti-CYP17A1, anti-CYP19A1, anti-PTGFS, anti-MMP1, anti-TIMP1, anti-CREB1, anti-AFT4, anti-FSHR, anti-TF, anti-VIM, anti-CYP11A1, and anti-LHCGR (donated by Dr. Marco Bonomi, Cusano Milanino MI, Italy)^8,60^ diluted in TBS-T buffer (Supplementary Table 1). Subsequently, membranes were washed three times in TBS-T and incubated with anti-rabbit or anti-mouse secondary antibodies conjugated with horseradish peroxidase (Bio-Rad) diluted (Supplementary Table 1) in TBS-T for 1.5 h at room temperature. Afterward, membranes were washed three times in TBS-T. Immune complexes were visualized using Clarity ECL substrate (Bio-Rad) according to the manufacturer’s protocol and developed in the ChemiDoc™ Touch Imaging System (Bio-Rad). Only for MMP1 were the anti-GAPDH antibodies (Supplementary Table 1) used as a loading control. The optical density of the protein bands detected on membranes, and the intensity of the protein bands on the TGX Stain-Free gels was analyzed using Image Lab 6 software (Bio-Rad). The abundance of tested proteins was quantified and normalized to either the total protein content in each equivalent lane or GAPDH (for MMP1).

### Total RNA isolation and real-time PCRTotal RNA isolation and real-time PCR

RNA isolation and expression analysis were performed as previously described^68^. Briefly, total RNA was isolated from walls of preovulatory follicles using a mirVana microRNA Isolation Kit (Invitrogen, Thermo Fisher Scientific) and genomic DNA was removed by DNAse I (Invitrogen), according to the manufacturer’s instructions. The purity and concentration of isolated RNA were determined using spectrophotometry using NanoDrop 1000 (Thermo Fisher Scientific). RNA integrity was evaluated with microfluidic electrophoresis by Agilent Bioanalyzer 2100 (Agilent Technologies, Santa Clara, CA, USA). Subsequently, RNA samples were reverse transcribed and amplified using the Taq-Man RNA-to-Ct1-Step Kit (Applied Biosystems, Thermo Fisher Scientific). The amplification reaction was prepared as follows: 0.25 μL TaqMan RT Enzyme Mix (40 ×), 5 μL TaqMan RT-PCR Mix (2 ×), 0.5 μL TaqMan Gene Expression Assay (20 × , Supplementary Table 3), 1.25 µL RNase-free water, and 5 ng of RNA. Real-time PCR was performed using a 7900 HT Real-Time PCR System (Applied Biosystems) in the following conditions: 48 °C for 15 min, 95 °C for 10 min, followed by 45 cycles of 15 s at 95 °C and 1 min at 60 °C. The real-time PCR Miner Software^65^ was used to estimate the mean PCR amplification efficiency and cycle threshold (Ct) values for each gene. The NormFinder algorithm^4^ was used to select the most stable reference among three tested genes: beta-actin (ACTB), glyceraldehyde 3-phosphate dehydrogenase (GAPDH), and hypoxanthine–guanine phosphoribosyltransferase (HPRT1).

### Statistical analysis

Statistica 13 (Cracow, Poland) was used to perform the statistical analysis. Two-way ANOVA and *post-hoc* Tukey test were used to determine (1) the content of steroid hormones, PGE_2_, and PGFM in the follicular fluid; (2) changes of mRNA expression in the walls of preovulatory follicles; (3) changes of protein expression in the walls of preovulatory follicles. Two main effects: maturity (MAT) and treatment (HORMONE), as well as interaction (MAT x HORMONE) are presented when statistically significant. Logarithmic transformation of the data was performed for non-normally distributed samples. All numerical data were expressed as mean ± standard error of the mean (SEM), and differences were considered statistically significant at *P* < 0.05.

Statistical analysis of changes in protein abundance in 2D-DIGE was performed using the Biological Variance Module of DeCyder Differential In-Gel Analysis version 5.02 software. For the PMF and MS/MS ion search, statistically significant (*P* ≤ 0.05) matches by Mascot were regarded as correct hits.

## Results

### hCG and GnRH-A challenge do not affect the number of visible follicles on ovaries

In prepubertal (Supplementary Fig. 1A) and mature (Supplemental Fig. 1B) gilts, the number of small and middle (≤ 6), as well as preovulatory follicles (6–8 and > 8 mm) did not differ between hCG- and GnRH-A-treated animals.

### Hormonal milieu of the follicular fluid is affected by sexual maturity or hormonal treatment (hCG or GnRH-A)

**Figure 1:**
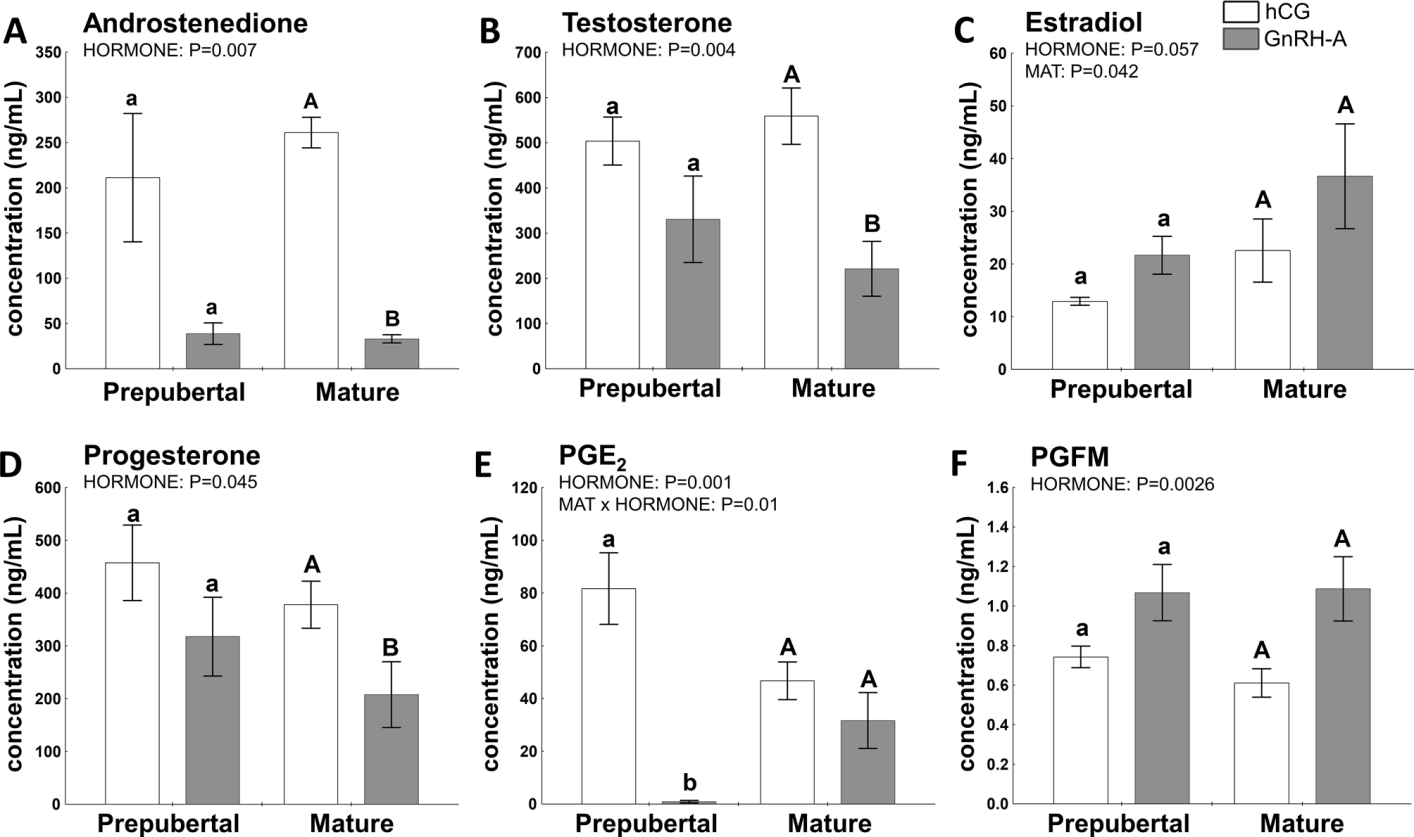
Hormonal milieu of the follicular fluid is affected by either sexual maturity status (prepuberty or maturity; MAT) or hormonal (hCG and GnRH-A; HORMONE) treatment in gilts. The follicular fluid A_4_ (**A**), T (**B**), E_2_ (**C**), P_4_ (**D**), PGE_2_, (**E**) and PGFM (**F**) is shown for prepubertal and mature gilts. Data are presented as mean ± SEM (n = 5–6 per group). Data were analyzed using two-way ANOVA and Tukey *post-hoc* test. Means with different superscripts differ significantly (small letters—prepubertal gilts, capital letters—mature gilts; *P* < 0.05).

Hormonal treatment affected A_4_ (*P* = 0.007), T (*P* = 0.004), and *P*_4_ (*P* = 0.045) levels in follicular fluid in challenged gilts (Fig. 1A, B, and D, respectively). Additionally, A_4_, T and *P*_4_ levels were significantly higher in hCG- *vs.* GnRH-A-treated mature gilts *(P* < 0.05).

Sexual maturity affected (*P* = 0.042) and hormonal treatment tended to affect (*P* = 0.057) E_2_ concentration in follicular fluid of treated gilts (Fig. 1C). Moreover, maturity and GnRH-A challenge decreased P_4_/E_2_ ratio (*P* = 0.045 and *P* = 0.0014, respectively), maintaining the preovulatory estrogenic status of follicles in both mature and prepubertal GnRH-A-treated gilts. The hormonal treatment significantly affected both androgens/estradiol ratios (T/E_2_ and A_4_/E_2_; *P* = 0.0002, and *P* = 0.0006, respectively) (Supplementary Table 4).

P_4_ concentration in follicular fluid was significantly correlated with A_4_ levels (r = 0.9007, *P* < 0.0001), T (r = 0.5484, *P* = 0.029), and PGE_2_ (r = 0.5258, *P* = 0.049), but P_4_/A_4_ ratio increased three to fivefold in GnRH-A—*vs.* hCG-treated gilts, as an effect of hormone (*P* = 0.006; Supplementary Table 4). PGE_2_ concentration in follicular fluid was also influenced by hormonal treatment (*P* = 0.001; MAT × HORMONE interaction, *P* = 0.01; Fig. 1E) and was 80-fold lower in GnRH-A-treated prepubertal gilts (*P* < 0.0025). The effect of hormonal treatment was also noticed for PGFM levels (*P* = 0.0026), which were twofold higher in GnRH-A- than in hCG-challenged mature gilts (*P* = 0.06; Fig. 1F).

### hCG/GnRH-A-treatment and sexual maturity lead to molecular changes in follicular walls of ovarian follicles

#### Factors related to progesterone, androgen, and estrogen synthesis

**Figure 2:**
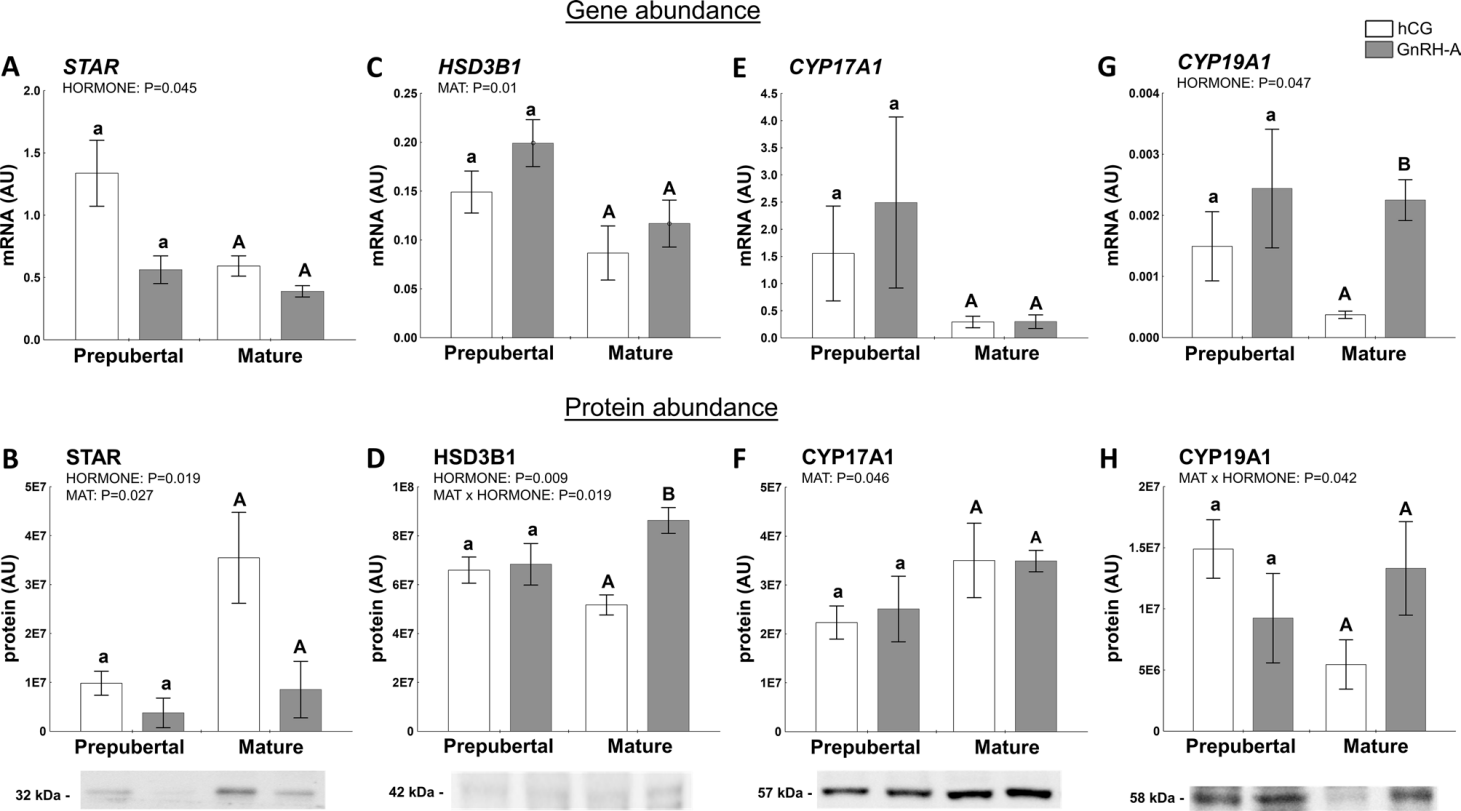
Hormones (hCG and GnRH-A; HORMONE) and sexual maturity status (prepuberty or maturity; MAT) change abundance of factors related to production of androgens and estrogens in ovarian follicles of prepubertal and mature gilts. The expression of STAR (**A**, **B**), HSD3B1 (**C**, **D**), CYP17A1 (**E**, **F**), and CYP19A1 (**G**, **H**) in prepubertal and mature gilts was evaluated. Gene expression was normalized to the geometric mean of ACTB and GAPDH (AU), identified as the best reference genes by NormFinder algorithm. Protein levels were normalized to total protein content (AU) using TGX Stain-Free gel technology (**B**, **D**, **F**, **H**). Uncropped blots are presented in Supplementary Fig. 3A online. Data were analyzed using two-way ANOVA with Sidak multiple comparison (mRNA) or Tukey (protein) *post-hoc* tests and are presented as mean ± SEM (n = 5–6 per group). Means with different superscripts differ significantly (small letters—prepubertal gilts, capital letters—mature gilts; *P* < 0.05). AU—arbitrary units.

Steroidogenic acute regulatory protein (STAR) was selected, as it plays a key role in the acute regulation of steroid hormone synthesis. In particular, it controls cholesterol entry into the mitochondria and limits steroidogenesis to the follicle^32^. Hormonal treatment affected STAR mRNA and protein abundance in the follicle (*P* = 0.045 and *P* = 0.019, Fig. 2A and B, respectively). However, the sexual maturity effect was only noticed for the STAR protein (*P* = 0.027; Fig. 2B). Interestingly, STAR protein abundance in follicular walls was positively correlated with T concentration in follicular fluid (r = 0.04971, *P* = 0.0036).

The abundance of hydroxy-delta-5-steroid dehydrogenase 3 beta- and steroid delta-isomerase 1 (HSD3B1) mRNA, an enzyme involved in P_4_ synthesis^39^, was affected by sexual maturity (*P* = 0.01; Fig. 2C), whereas hormonal treatment strongly affected its protein levels (*P* = 0.009; MAT × HORMONE interaction, *P* = 0.019; Fig. 2D), reaching significance in mature gilts (*P* < 0.014).

Abundance of CYP17A1 mRNA (Cytochrome P450 Family 17 Subfamily A Member 1), an enzyme involved in androstenedione synthesis^39^, was not changed (Fig. 2E), but sexual maturity positively affected its protein level (*P* = 0.046; Fig. 2F). In addition, CYP17A1 protein levels were negatively correlated with other proteins tested: TIMP1 (r =  − 0.7420; *P* = 0.001) and CYP19A1 (r =  − 0.5542; *P* = 0.021).

In follicular walls, the mRNA abundance of an enzyme responsible for a key step in biosynthesis of estrogens—cytochrome P450 family 19 subfamily a member 1 (CYP19A1) was affected by hormonal treatment (*P* = 0.047; Fig. 2G). MAT × HORMONE interaction was identified for the CYP19A1 protein (*P* = 0.042; Fig. 2H). In addition, CYP19A1 protein levels were correlated with other proteins: TIMP1 (r = 0.6985; *P* = 0.02), PTGFS (r =  − 0.5311; *P* = 0.028), and CYP17A1 (r =  − 0.5542; *P* = 0.021).

Sexual maturity did not affect LHCGR mRNA in follicles (Supplementary Fig. 2A), but it affected its protein levels (*P* = 0.041; Supplementary Fig. 2) in the follicular walls of hCG- and GnRH-A-treated gilts. Interestingly, abundance of LHCGR was positively correlated with follicular E_2_ level for both mRNA (r = 0.611, *P* = 0.016) and protein (r = 0.7359, *P* = 0.003). In addition, the LHCGR protein correlated with other proteins tested: PTGFS (r = 0.4767; *P* = 0.045) and ATF4 (r =  − 0.4270; *P* = 0.4362).

For FSHR mRNA abundance, only a tendency was noticed for HORMONE (*P* = 0.06; Supplementary Fig. 2B), while it reached significance for protein (*P* = 0.02; Supplementary Fig. 2D). In addition, FSHR protein was negatively correlated with PTGFS protein abundance (r =  − 0.554; *P* = 0.021).

#### Factors related to production and action of PGs

**Figure 3:**
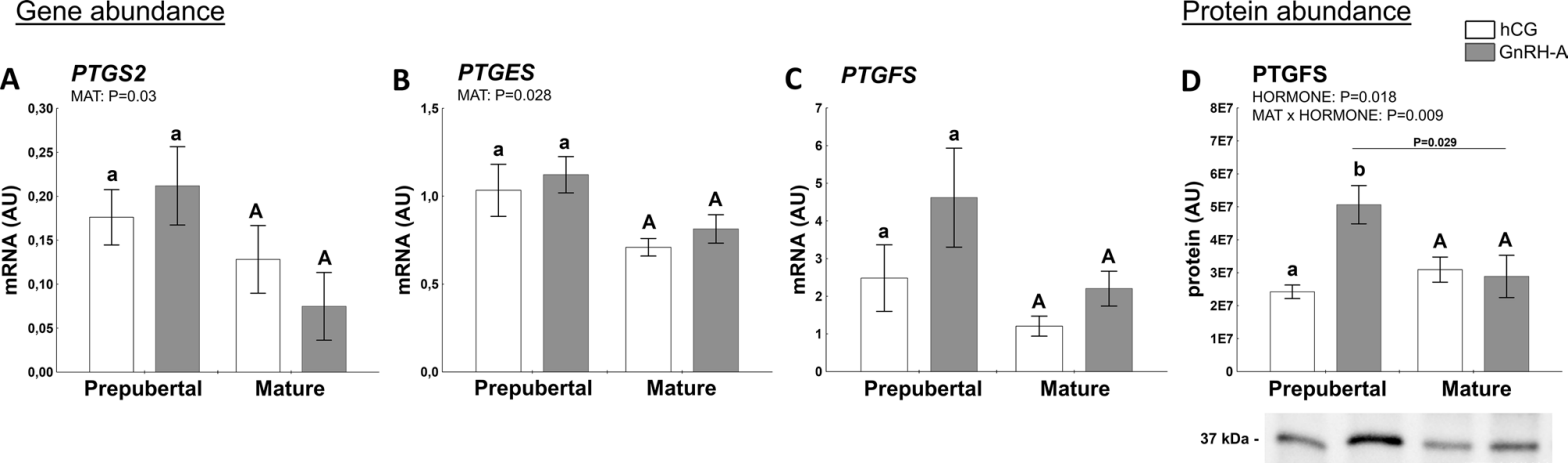
Hormones (hCG and GnRH-A) and sexual maturity status (prepuberty or maturity; MAT) affect abundance of factors involved in prostaglandin synthesis in ovarian follicles of prepubertal and mature gilts. The abundance of PTGS2 mRNA (**A**), PTGES mRNA (**B**), and PTGFS mRNA/protein (**C**, **D**) in prepubertal and mature gilts was evaluated. Gene expression was normalized to the geometric mean of ACTB and GAPDH, identified as the best reference genes by NormFinder algorithm. Protein levels were normalized to total protein content using TGX Stain-Free gel technology (**D**). Uncropped blots are presented in Supplementary Fig. 3B online. Data were analyzed using two-way ANOVA with Sidak multiple comparison (mRNA) or Tukey (protein) *post-hoc* tests and are presented as mean ± SEM (n = 5–6 per group). Means with different superscripts differ significantly (small letters—prepubertal gilts, capital letters—mature gilts; *P* < 0.05). Line with a P value denote significant differences between prepubertal and mature gilts.

PGs play a crucial role in the development and ovulation of preovulatory follicles^18,20^. Thus, both mRNA and protein abundance of the selected components of the PGE_2_ and PGF_2α_ synthesis pathways were evaluated in follicular walls of prepubertal and mature gilts challenged with hormones (hCG or GnRH-A). Prostaglandin-endoperoxide synthase 2 (PTGS2, known also as COX2) is involved in the conversion of arachidonic acid to prostaglandin H2 (PGH2), which is next converted to PGE_2_ and PGF_2α_ or prostacyclin and thromboxane. Sexual maturity affected mRNA abundance of PTGS2 in the follicular walls of challenged gilts (*P* = 0.03; Fig. 3A). The mRNA abundance of PGE_2_ synthase (PTGES), was also affected by maturity (*P* = 0.028; Fig. 3B). The mRNA abundance of PGF_2α_ synthase (PTGFS) did not differ in follicular walls (Fig. 3C). However, hormonal treatment strongly affected PTGFS protein expression (*P* = 0.018; MAT × HORMONE interaction, *P* = 0.009; Fig. 3D). Interestingly, a 2.5-fold higher abundance of the PTGFS protein was indicated in prepubertal gilts challenged with GnRH-A compared with mature counterparts (*P* = 0.029). It was also significantly increased when compared to hCG-treated prepubertal gilts (*P* = 0.0096). A negative correlation was observed between the PTGFS mRNA abundance and the P_4_ concentration in follicular fluid (r =  − 0.6398; *P* = 0.046).

#### Factors related to the transformation of preovulatory follicles in luteal tissue

**Figure 4:**
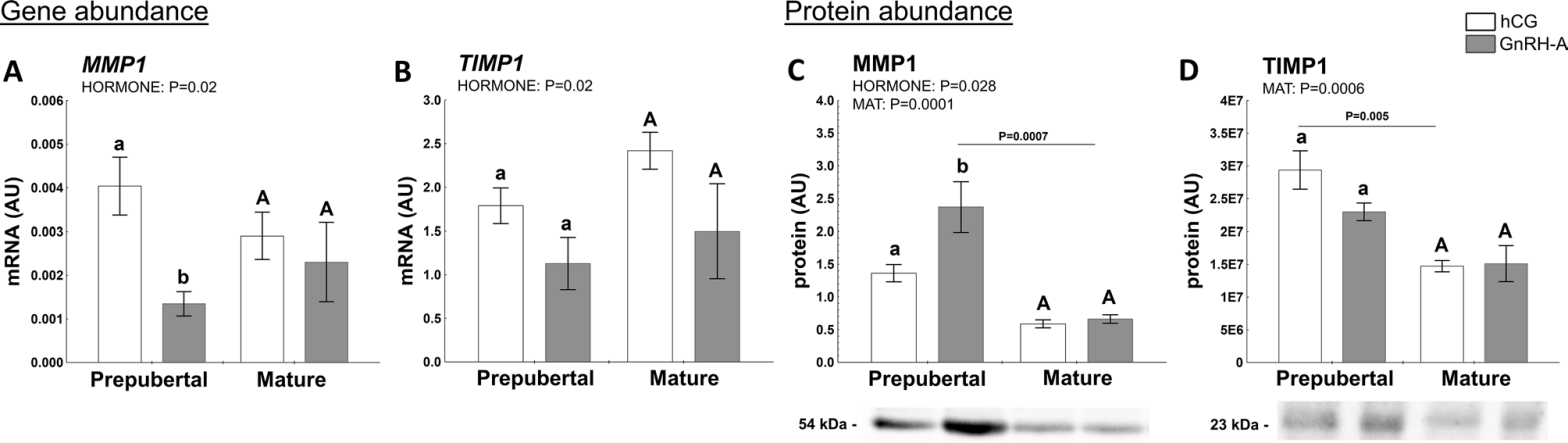
Hormones (hCG and GnRH-A; HORMONE) and sexual maturity status (prepuberty or maturity; MAT) affect abundance of extracellular matrix regulators in ovarian follicles of prepubertal and mature gilts. The abundance of MMP1 (**A**, **C**) and TIMP1 (**B**, **D**) in prepubertal and mature gilts was evaluated. Gene expression was normalized to the geometric mean of ACTB and GAPDH (AU), identified as the best reference genes by NormFinder algorithm. Protein levels were normalized to either total protein content (AU) using TGX Stain-Free gel technology (**D**) or GAPDH loading control (**C**). Uncropped blots are presented in Supplementary Fig. 3C online. Data were analyzed using two-way ANOVA with Sidak multiple comparison (mRNA) or Tukey (protein) *post-hoc* tests and are presented as mean ± SEM (n = 5–6 per group). Means with different superscripts differ significantly (small letters—prepubertal gilts, capital letters—mature gilts; *P* < 0.05). Line with a *P *value denote significant differences between prepubertal and mature gilts. AU – arbitrary units.

The MMP-TIMP system is involved in the proteolytic network of follicular development and rupture of the follicle wall with successful ovulation^26^. In preovulatory follicles, the amounts of collagenases (matrix metalloproteinase [MMP]1 and MMP2) arise. MMPs are inhibited by tissue-specific inhibitors (TIMPs; e.g., TIMP1 and TIMP2) and limit follicular wall destruction^45^. Thus, both mRNA and protein abundance of selected MMP-TIMP system components were evaluated in follicular walls of prepubertal and mature gilts challenged with hormones (hCG or GnRH-A). MMP1 mRNA abundance in follicular walls was affected by hormonal treatment (*P* = 0.02) and remained threefold higher in hCG- than GnRH-A-treated prepubertal gilts (*P* = 0.017, Fig. 4A). By contrast, MMP1 protein expression was significantly higher in prepubertal GnRH-A- than hCG-challenged gilts (*P* < 0.05) and in addition to hormonal treatment (*P* = 0.028) was strongly affected by sexual maturity (*P* = 0.0001, Fig. 4C. Expression of TIMP1 mRNA was also affected by hormonal treatment (*P* = 0.02; Fig. 4B). As for MMP1, TIMP1 protein levels in follicular walls were strongly affected by sexual maturity (*P* = 0.0006; Fig. 4D). In addition, TIMP1 protein abundance was twofold higher in follicles of hCG-treated prepubertal *vs.* mature gilts (*P* = 0.005). TIMP1 protein abundance was also positively correlated with MMP1 (r = 0.5515; *P* = 0.022) and CYP19A1 (r = 0.6985; *P* = 0.002) and negatively correlated with CYP17A1 (r =  − 0.7420; *P* = 0.001) proteins.

#### Factors related to transcription regulation of ovarian function

**Figure 5:**
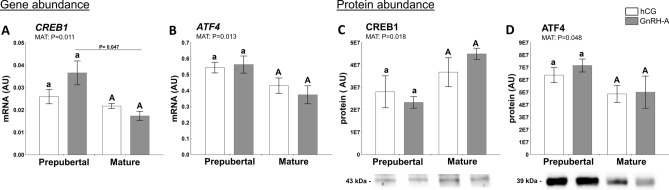
Hormones (hCG and GnRH-A; HORMONE) and sexual maturity status (prepuberty or maturity; MAT) affect abundance of transcription factors governing in ovarian follicles of prepubertal and mature gilts. The abundance of CREB1 (**A**, **C**) and ATF4 (**B**, **D**) in prepubertal and mature gilts was evaluated. Gene expression was normalized to the geometric mean of ACTB and GAPDH (AU), identified as the best reference genes by NormFinder algorithm. Protein levels were normalized to total protein content (AU) using TGX Stain-Free gel technology (**C**, **D**). Uncropped blots are presented in Supplementary Fig. 3C online. Data were analyzed using two-way ANOVA with Sidak multiple comparison (mRNA) or Tukey *post-hoc* (protein) tests and are presented as mean ± SEM (n = 5–6 per group). Means with different superscripts differ significantly (small letters—prepubertal gilts, capital letters—mature gilts; P < 0.05). Line with a P value denote significant differences between prepubertal and mature gilts. AU—arbitrary units.

The cAMP response element-binding protein (CREB1) and activating transcription factor 4 (ATF4, known as CREB2) play a vital role in the control of ovarian steroidogenesis^17^. Thus, mRNA and protein abundance of both cellular transcription factors was evaluated in follicular walls of prepubertal and mature gilts challenged with hormones (hCG or GnRH-A). Maturity affected CREB1 mRNA (*P* = 0.011; Fig. 5A) and protein abundance (*P* = 0.018; Fig. 5C). The abundance of AFT4 mRNA (*P* = 0.013; Fig. 5B) and protein (*P* = 0.048; Fig. 5D) was also affected by maturity. In addition, ATF4 protein levels were positively correlated with HSD3B1 (r = 0.5652; *P* = 0.018) and negatively correlated with PTGFS (r =  − 0.4854; *P* = 0.048) protein levels.

### Proteome of follicular walls of prepubertal and mature gilts is affected by hCG- or GnRH-A-treatment

**Figure 6:**
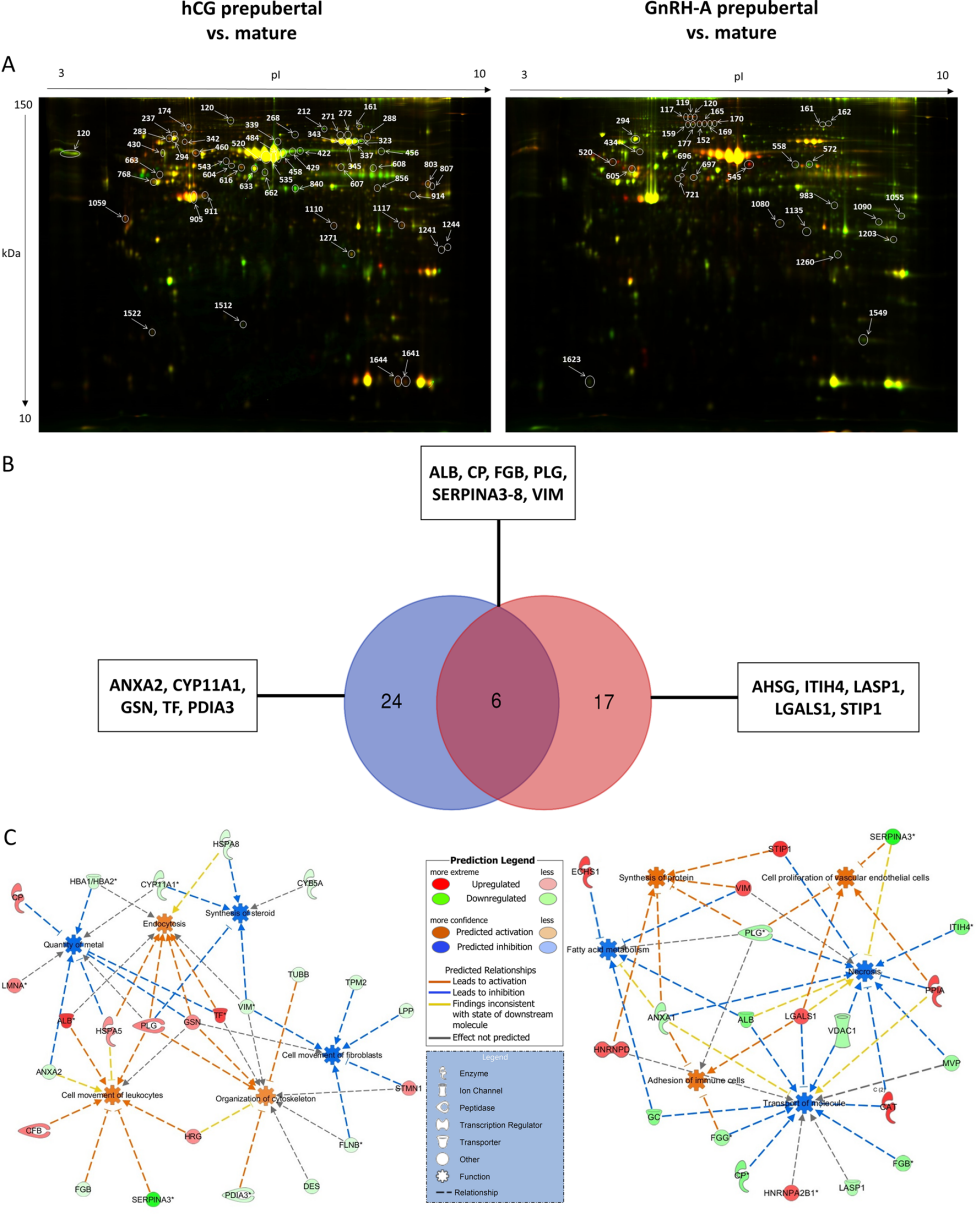
(**A**) Representative 2D-DIGE gel image of differentially abundant proteins in follicular walls of: (left panel) hCG-treated prepubertal (Cy3, green) *vs.* mature gilts (Cy5, red) and (right panel) GnRH-A-treated prepubertal (Cy3, green) *vs.* mature gilts (Cy5, red). Spot numbers correspond to the proteins which were identified by MALDI- TOF/TOF analysis. (**B**) Venn diagram shows hCG (blue) and GnRH-A (red) treatment affected proteins in prepubertal and mature gilts. (**C**) Molecular pathway enriched by differentially abundant proteins identified in follicular walls of treated gilts. Red and green colors depict an increase or decrease, respectively, in abundance of the proteins in follicular walls of treated gilts. The color intensity of nodes indicates a fold change increase or decrease associated with a particular protein. Direct and indirect interactions are indicated by solid, and dash lines, respectively.

To evaluate how hormonal treatment (GnRH-A and hCG) and sexual maturity affect prepubertal and mature gilts, we used 2D-DIGE to assess differentially abundant proteins in follicular walls. Figure 6A presents representative 2D gel images depicting significantly altered protein spots in follicular walls of hCG- or GnRH-A-treated prepubertal gilts compared with mature counterparts. The corresponding identified proteins along the fold change in abundance are listed in Supplementary Tables 5 and 6, respectively.

In total, 30 differentially abundant proteins were identified in the follicular walls of hCG-treated prepubertal *vs.* mature gilts (Fig. 6B). Ten proteins were upregulated, including transferrin (TF; FC = 1.69; *P* = 0.0045), ceruloplasmin (CP; FC = 1.44; *P* = 0.03), complement factor B (CFB; FC = 1.38; *P* = 0.01), gelsolin (GSN; FC = 1.22; *P* = 0.015), and stathmin 1 ( STMN1; FC = 1.2; *P* = 0.049). Twenty proteins were downregulated, including serpin family A member 3 (SERPINA3-8; FC =  − 7.51; *P* = 0.045), cytochrome P450 family 11 subfamily A member 1 (CYP11A1; FC =  − 1.66; *P* = 0.018), hemoglobin subunit alpha (HBA1; FC =  − 1.57; *P* = 0.0033), annexin A2 (ANXA2; FC =  − 1.55; *P* = 0.0088), and vimentin (VIM; FC =  − 1.35; *P* = 0.022). The top molecular and cellular functions associated with proteins showing higher abundance in the follicular walls of hCG-treated prepubertal gilts were cellular movement (nine proteins), cellular function and maintenance (eight proteins), and cellular assembly and organization (ten proteins). On the other hand, the most significantly affected functions in follicular walls of hCG-treated mature gilts were molecular transport (nine proteins), cellular movement (six proteins), and lipid metabolism (five proteins; Fig. 6C, Table 1).

**Table 1:** Functional analysis of differentially abundant proteins in follicular walls of prepubertal and mature gilts treated with either hCG or GnRH-A.

hCG		GnRH-A	
Top molecular and cellular function	Molecules	Top molecular and cellular function	Molecules
Cellular Movement	ALB, ANXA2, CFB, FGB, GSN, HRG, HSPA5, PLG, SERPINA3	Proteins Synthesis	ANAXA1, HNRNPD, PLG, STIP1, VIM
Cellular Function and Maintenance	ALB, GSN, HBA1, HSPA5, HSPA8, PLG,TF, VIM	Cardiovascular System Development and Function	LGALS1, PLG, PPIA, SERPINA3
Cellular Assembly and Organization	DES, FLNB, GSN, HRP, PDIA3, PLG, STMN1, TF, TUBB, VIM	Cell to Cell Signaling and Interaction	ANXA1, FGG, LGALS1, PLG
Molecular Transport	ANXA2, CP, CYP11A1, GSN, HBA1, HSPA5, LMNA, PLG, TF	Molecular Transport	ALB, ANXA1, CAT, CP, FGB, FGG, GC, HNRNPA2B1, HNRNPD, LASP1, LGALS1, MVP, PPIA, VDAC1
Cellular Movement	FLNB, GSN, LPP, STMN1, TPM2, VIM	Cell Death and Survival	ALB, ANXA1, CAT, ITIH4, LGALS1, MVP, PLG, PPIA, SERPINA3, STIP1, VDAC1, VIM
Lipid Metabolism	CYB5A, CYP11A1, HSPA8, PLG, VIM	Lipid Metabolism	ALB, ANXA1, ECHS1, GC, PLG, VIM

In GnRH-A-treated gilts, 23 differentially abundant proteins were detected in follicular walls of prepubertal *vs.* mature counterparts (Fig. 6B). Of these, eight proteins were upregulated: catalase (CAT; FC = 1.46; *P* = 0.013), stress-induced phosphoprotein 1 (STIP1; FC = 1,46; *P* = 0.013), peptidylprolyl isomerase A (PPIA; FC = 1.27; *P* = 0.0011), vimentin (VIM; FC = 1.23; *P* = 0.025), and galectin 1 (LGALS1; FC = 1.19; *P* = 0.018). Furthermore, 15 proteins were downregulated, including serpin family A member 3–8 (SERPINA3-8; FC =  − 3.28; *P* = 0.012), inter-alpha-trypsin heavy chain H4 (ITIH4; FC =  − 1.91; *P* = 0.003), ceruloplasmin (CP; FC =  − 1.83; *P* = 0.0047), plasminogen (PLG; FC =  − 1.35; *P* = 0.043) and LIM and SH3 protein 1 (LASP1; FC =  − 1.18; *P* = 0.016). The top molecular and cellular functions cataloged for prepubertal GnRH-A-treated gilts were protein synthesis (five proteins), cell-to-cell signaling and interaction (five proteins), and cardiovascular system development and function (four proteins), whereas their mature counterparts were molecular transport (fourteen proteins), cell death and survival (twelve proteins), and lipid metabolism (six proteins; Fig. 6C).

We also searched in silico for upstream regulators of differentially abundant proteins. In the hCG-treated gilts, TGFB1 (z-score: − 2.84; *P* = 3.04E-08) and IL-4 (z-score: − 2.19; *P* = 1.04E-02) were identified among upstream regulators. Both were marked as significantly inhibited regulators in mature hCG-treated gilts. In GnRH-A-treated gilts, among upstream regulators, EGFR (z-score: 1.78; *P* = 6.99E-06), ESR1 (z-score: 1.73; *P* = 4.67E-02) were identified as activated, and IL-6 (z-score: − 1.62; *P* = 2.81E-08) as inhibited.

A Venn diagram was created to identify common and characteristic proteins in follicular walls of hCG- and GnRH-A-treated gilts (Fig. 6B). Six proteins were common for both variants of treatment (ALB, PLG, CP, VIM, SERPINA3-8, and FGB), 24 were characteristic for hCG- and 17 for GnRH-A-treated gilts. Interestingly, molecular transport (z-score –2.41 and –1.93, respectively) and lipid metabolism (z-score − 1.96 and − 1.72, respectively) were identified among the top biofunctions for both hCG- or GnRH-A-treated gilts.

**Figure 7:**
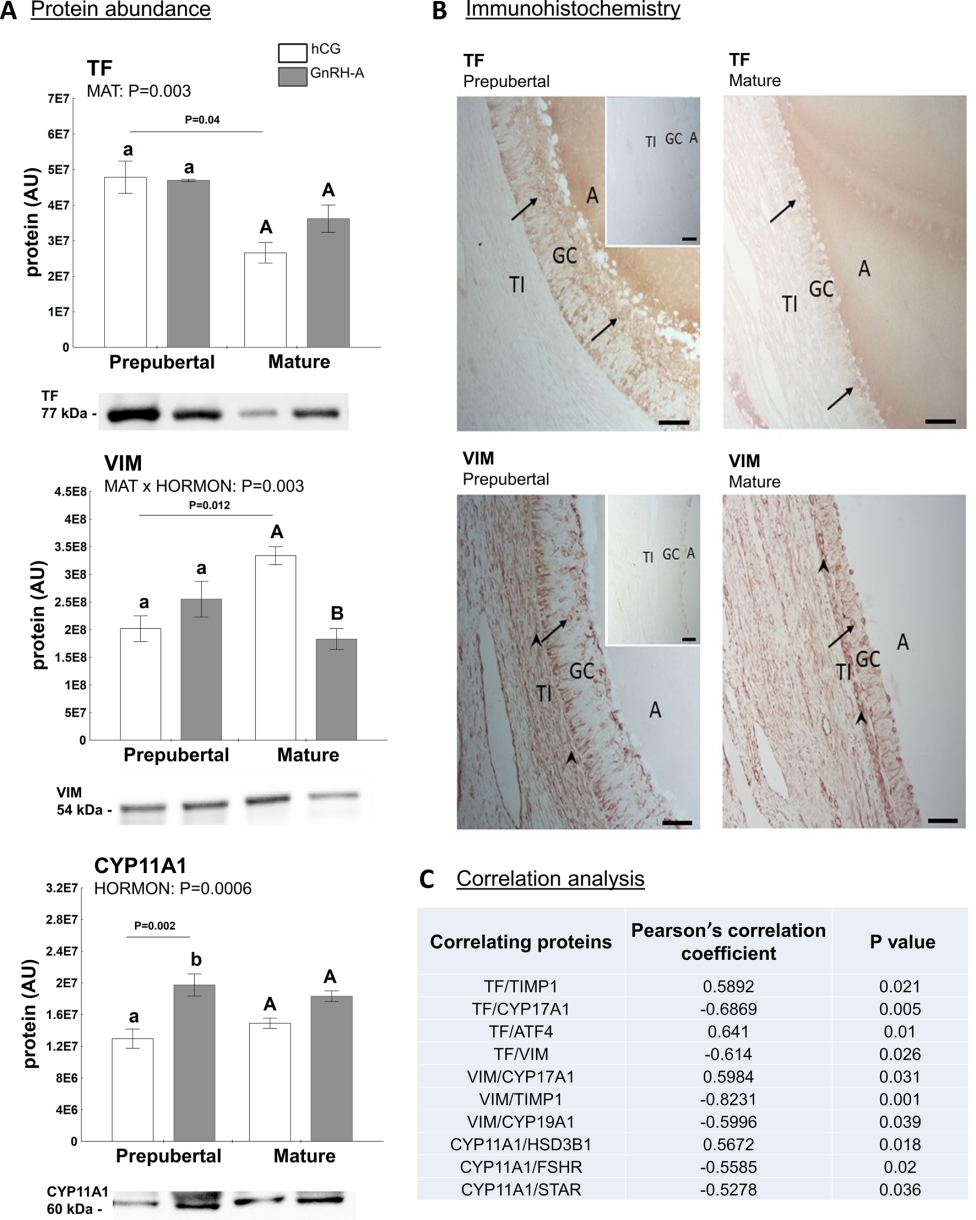
Abundance of proteins (TF, VIM and CYP11A1) in follicular walls of treated gilts, selected for validation of 2D-DIGE by Western blot (**A**) and immunohistochemistry (**B**). (**A**) Protein levels were normalized to total protein content (AU) using TGX Stain-Free gel technology. Uncropped blots are presented in Supplementary Fig. 3D online. Data were analyzed using two-way ANOVA with Tukey *post-hoc* tests and are presented as mean ± SEM (n = 5–6 per group). Means with different superscripts differ significantly (small letters—prepubertal gilts, capital letters—mature gilts; *P* < 0.05). Line with a P value denote significant differences between prepubertal and mature gilts. AU – arbitrary units. (**B**) TF and VIM immunostaining was performed in large antral follicles of prepubertal and sexually mature gilts. The arrow indicates positive staining in granulosa cells (GC); the arrowhead indicates positive staining in theca cells. Control sections in which the primary antibody was replaced by rabbit or mouse IgG were free from staining (insets). A—antrum, TI—*theca interna*; scale bars represent 50 µm. (**C**) Correlations between proteins selected for validation and other factor tested in follicular walls of treated gilts.

Three differentially abundant proteins—VIM (common for both treatments), TF (characteristic for hCG-treated), and CYP11A1 (characteristic for hCG-treated)—were further validated using Western blotting (Fig. 7). In addition to its abundance in our experimental setting, each protein was selected for further analysis due to its known function in ovarian physiology. Briefly, VIM and TF are essential proteins involved in the local regulation of ovarian function, whereas CYP11A1 is a key enzyme regulating steroidogenesis in follicles and commits cholesterol to the steroidogenic pathway^39^.

As presented in Fig. 7, the abundance of selected proteins was consistent with this obtained in the 2D-DIGE analysis. TF abundance was affected by sexual maturity (P = 0.003) and it was maintained higher in follicular walls of hCG-treated prepubertal *vs.* mature gilts (P = 0.04). No significant differences were observed in TF abundance in GnRH-A-treated gilts (Fig. 7A). Interestingly, TF protein abundance was positively correlated with TIMP1 (r = 0.5892; *P* = 0.021) and ATF4 (r = 0.641; *P* = 0.01) and negatively correlated with E_2_ (r =  − 0.553; *P* = 0.033), CYP17A1 (r =  − 0.6869; *P* = 0.005), and VIM (r =  − 0.614; *P* = 0.026) protein levels (Fig. 7C). MAT × HORMONE interaction was identified for VIM (*P* = 0.003). Further analysis showed that its abundance in mature gilts was higher after hCG then GnRH-A treatment (1.8-fold; *P* = 0.039) and when compared to prepubertal hCG-challenged gilts (*P* = 0.012; Fig. 7A). VIM protein abundance was positively correlated with CYP17A1 (r = 0.5984; *P* = 0.031) and E_2_ (r = 0.6531; *P* = 0.011) and negatively correlated with TIMP1 (r =  − 0.8231; *P* = 0.001), CYP19A1 (r =  − 0.5996; *P* = 0.039), and PGE_2_ (r =  − 0.5254; *P* = 0.037; Fig. 7C). Hormonal treatment clearly affected CYP11A1 protein expression (*P* = 0.0006) and its levels were higher in the follicular walls of prepubertal GnRH-A- than hCG-treated gilts (*P* = 0.002; Fig. 7A). Interestingly, this enzyme protein abundance was negatively correlated with P_4_ (r =  − 0.5471; *P* = 0.019), A_4_ (r =  − 0.5877; *P* = 0.017), T (r =  − 0.6407; *P* = 0.003), and PGE_2_ (r =  − 0.6058; *P* = 0.01) levels in follicular fluid. Also, negative correlation for STAR (r =  − 0.5278; *P* = 0.036), FSHR (r =  − 0.5585; *P* = 0.02) and positive for HSD3B1 (r = 0.5672; *P* = 0.018; Fig. 7C) was noticed.

### Transferrin and vimentin are expressed in granulosa and theca cells in large antral follicles of prepubertal and mature gilts

As there was no data about the localization of TF and VIM in ovarian follicles in pigs, we performed immunohistochemical localization of both factors in antral follicles (Fig. 7B). Immunohistochemistry revealed positive staining for TF in the cytoplasm of granulosa cells of large antral follicles obtained from prepubertal and mature gilts. Although we did not quantify staining, it seems that the immunostaining intensity of TF was associated with sexual maturity, and the stronger intensity of TF staining was found in the large antral follicles of premature gilts. In all examined sections, the VIM protein was detected in the cytoplasm of both granulosa and theca cells of large antral follicles obtained from prepubertal and mature gilts. Similar to TF staining, the intensity of VIM immunoreaction seemed to be associated with sexual maturity. However, a likely stronger VIM staining intensity was found in the large antral follicles of mature gilts.

## Discussion

Optimal reproductive performance is managed by different strategies to meet the high-performance expectations of modern farms. Hormones have been used to control the reproductive functions of sows and gilts in different protocols to optimize management practices and improve overall pork production efficiency^37^. Pharmaceuticals, such as progesterone analogs and gonadotropins, are commonly used in a number of countries in practice to aid the reproductive performance of gilts and sows. In this study, we provide evidence for the different endocrine properties of endogenous LH and exogenous hCG in the control of preovulatory follicles in pigs. Our data indicate that gonadotropins used in managing the reproductive performance of sows and gilts affect endocrine and molecular milieus of preovulatory follicles, besides depending on sexual maturity.

The endocrine milieu of preovulatory follicles was significantly affected by the hCG trigger, as higher levels of P_4,_ androgens (A_4_ and T), and PGE_2_ were observed, accompanied by an increased abundance of STAR protein in mature pigs. In addition, the following hormone ratios, P_4_/E_2_, T/E_2_, and A_4_/E_2_, were higher in hCG- than GnRH-A-treated prepubertal and mature gilts. The P_4_/E_2_ ratio in the follicular fluid of prepubertal and mature GnRH-A-treated gilts falls into the category of preovulatory (estrogenic) follicles^67^. Higher P_4_/E_2_ ratio in hCG- compared with GnRH-A-treated gilts indicates an accelerated luteinization process. Also, much higher androgens/E_2_ ratios suggest androgenization of preovulatory follicles in hCG-treated gilts. Interestingly, hCG maintained a higher PGE_2_ concentration in the follicular fluid of prepubertal pigs. Similar observations of hCG-induced PGE_2_ production were made for human granulosa cells^54^, suggesting the possible relevance of our work to human reproductive medicine.

The second ovulation trigger—GnRH-A—affected HSD3B1, CYP11A1, and PTGFS protein abundance. Aromatase declines between days 18 and 20 of the estrous cycle despite a rise of E_2_ in the follicular fluid, and the availability of androgen substrate appears to be critical for maintaining E_2_ synthesis^23^. E_2_ concentration in follicular fluid followed the pattern of CYP17A1 rather than CYP19A1 protein abundance, and a significant correlation between CYP17A1 protein and E_2_ concentration in follicular fluid was found, as in our recent study^68^. Considering these findings, the concept that CYP17A1 is the rate-limiting enzyme of follicular estrogen synthesis in pigs^23^ is even more probable. Interestingly, neither GnRH-A nor hCG triggers directly affected CYP19A1 protein abundance, although mRNA levels were decreased in hCG- compared with GnRH-A-treated mature pigs. In pigs, unlike other animal species, CYP11A1 is present in both the granulosa and theca layers, and its levels appear to be equal at least at mRNA levels^13^.

Our studies confirm a strong correlation of LHCGR protein expression with follicular E_2_ and PGFS protein levels. In addition, PGFM concentration in follicular fluid affected by hormonal treatment followed PTGFS protein abundance in follicles of prepubertal and mature gilts. Sexual maturity affected MMP1 and TIMP-1 proteins involved in the luteinization process^44^, but GnRH-A and hCG treatment also altered MMP1 protein expression in prepubertal gilts. It strongly suggests that the proteolytic mechanism in the preovulatory follicle of prepubertal gilts is susceptible to the specific post-LHCGR signaling activated by LH and hCG. The maturity also ensured the stable, hCG- and GnRH-A-stimuli-independent expression of transcription factors, such as CREB1 and ATF4. Maturity affected CREB1 and ATF4 protein concentration in follicular walls of hCG- and GnRH-A-treated groups. Both observations agree with our recent report^68^. CREB1 and AFT4 play a vital role in the control of ovarian steroidogenesis via cAMP signaling. On the other hand, AFT4 is involved in prostaglandin synthesis by binding to the PTGES2 promoter and increasing PGE_2_ production in response to hCG in human granulosa cells^17^.

Our study is the first to report such a broad proteomic change in preovulatory follicles of prepubertal and mature gilts evoked by either exogenous hCG or native LH, released by GnRH-A. We found that hCG or GnRH-A administration altered the abundance of several proteins that are associated with lipid metabolism, extracellular matrix (ECM) remodeling, folding of proteins, and cell proliferation/survival and cellular signaling. Proteins associated with ECM remodeling were upregulated in the follicular walls of mature hCG-treated gilts, including ACTB, TPM2, VIM, and SERPINA3. Actin binding proteins play a vital role in the formation of follicles before ovulation, which includes cell signaling and growth, as well as the maintenance of cell shape and differentiation^49^. Changes in the expression of VIM and TPM2 mRNA upon gonadotropin stimulation were also reported in human granulosa cells^24^. Interestingly, SERPINA3 (alpha-1-antichymotrypsin), belonging to the protease inhibitor family^5^ and acute phase proteins^36^, showed remarkably higher protein abundance in mature than prepubertal hCG- or GnRH-A-treated gilts. SERPINA3 activates inflammatory cytokines, remodels tissues, and prevents follicular cell apoptosis^12,62^. VIM is a cytoskeletal intermediate filament protein that is vital for organelle transport, cell migration, and proliferation, as well as the transfer of free cholesterol from the cytoplasm to mitochondrial outer membrane, thus forming a bridge between cholesterol and mitochondria^41,53^. Interestingly, the VIM protein was positively correlated with E_2_ and CYP17A1 and negatively correlated with CYP19A1, TIMP1, TF, and PGE_2_ levels, suggesting its crucial but yet unknown roles in follicular development and function.

The abundance of GSN, PLG, and TF proteins, which are involved in ovarian follicle function, was higher in the follicular walls of prepubertal than in mature hCG-treated gilts. GSN modulates estrogen receptor function and aromatase expression^52^. PLG is converted to plasmin during ovulation, which in turn decreases the follicular tensile strength at ovulation^6^. Apart from its iron-binding properties, TF plays a vital role in the local regulation of ovarian function, inhibiting aromatase activity^40^ or differentiation^64^ of rat granulosa cells. TF also inhibits FSH-stimulated aromatase activity in porcine granulosa cells^22^. However, no correlation was noted between TF and CYP19A1 protein in follicular walls, but a negative correlation was observed between CYP17A1 protein expression and E_2_ concentration in follicular fluid. Thus, the role of TR in the porcine granulosa layer requires further investigation.

The other class of proteins with high expression in the follicular walls of mature hCG-treated gilts was associated with the efficient folding of proteins and maintaining protein homeostasis in cells. We observed that the abundance of HSPA8 and SERPINH1 (HSP47) was significantly higher in follicular walls of mature hCG-treated gilts. HSPs can prevent the incorrect folding of proteins and possess antiapoptotic properties and oxidative stress^30,35,38^. HSPA8, a chaperone protein, plays a key role in regulation of steroid hormone function by modulating their receptor activity, including estrogen, progesterone, and androgen receptors^50^^,^^55^. HSP47 is essential for the correct folding of procollagen^33^. Increased collagen and HSP47 expression are implicated in the pathogenesis of fibrotic diseases and cyst formation^57^. Our previous study suggested that HSPs have a protective role in preovulatory follicles during differentiation of estrogen-producing follicular cells to progesterone-producing luteal cells^41^.

Proteins associated with lipid metabolism had a higher abundance in the follicular walls of mature hCG- or GnRH-A-treated gilts. Among them, the redox protein CYB5A is involved in lipid biosynthesis, delivering electrons to microsomal desaturases that synthesize steroids and fatty acids^59^. It was shown to have a specific function in porcine granulosa and theca layers^15^ and intraovarian androstenedione production^46^. We also observed an increase in the abundance of ANXA1 and GC in mature animals. ANXA1 is a calcium and phospholipid-binding protein of the annexin superfamily^56^, whose expression is stimulated by 17-beta-estradiol and is involved in cell growth, differentiation, apoptosis, and membrane fusion^34^. GC has essential physiological functions, including vitamin D transport and storage^61^. Grzesiak and coworkers^27^ reported that vitamin D regulates follicular P_4_ and E_2_ synthesis, and suggested its essential role in follicular development in mature gilts. The altered abundance of proteins related to lipid metabolism in the follicular walls of mature hCG- and GnRH-traded gilts suggest, respectively, early luteinization of preovulatory follicles and positive stimulation of follicular growth.

Among proteins identified by 2D-DIGE, only one enzyme involved in the steroidogenic pathway was identified, i.e., CYP11A1. Studies have shown CYP11A1 expression in theca cells of antral follicles, which increased during porcine follicle maturation^25^. However, porcine follicles exposed to LH preovulatory surge did not show a decline in CYP11A1 mRNA^2^. The higher CYP11A1 protein abundance affected by native LH (GnRH-A) seems to confirm these earlier studies. Interestingly, CYP11A1 protein was positively correlated with HSD3B1 involved in synthesis P_4_, but negatively with STAR protein, which mediates cholesterol entry into mitochondria for conversion to pregnenolone by CYP11A1. PKA signals induced by the natural LH surge or GnRH-A released LH are the initial stimuli that increase CYP11A1 abundance during follicle-to-corpus luteum transition^31^.

**Figure 8:**
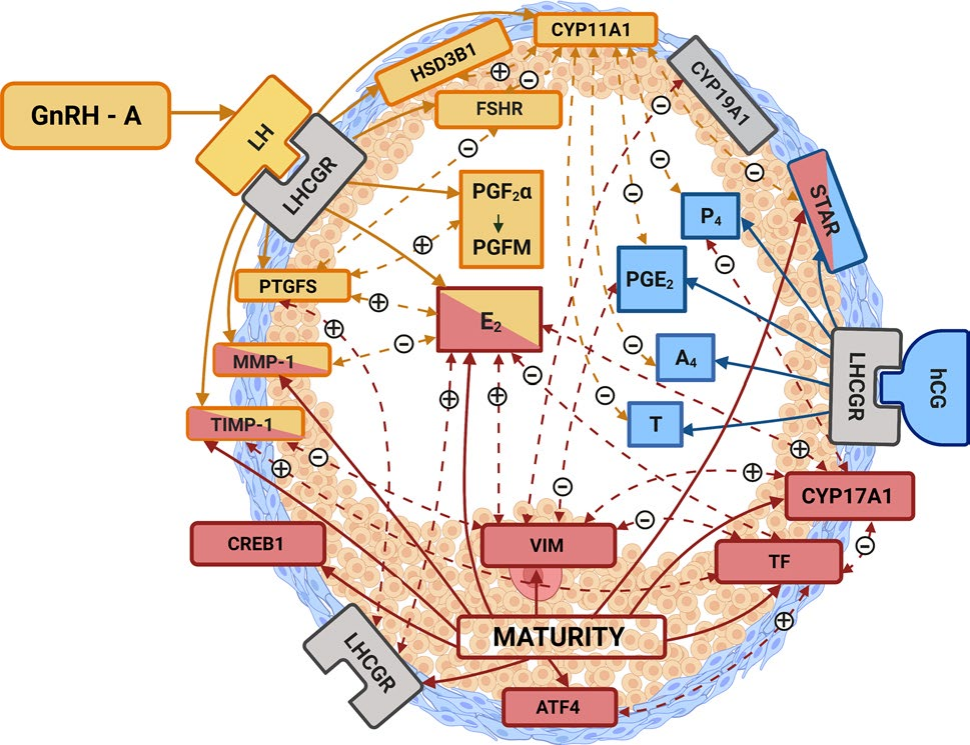
Illustrative summary of two ovulation stimuli: hCG, GnRH-A and the sexual maturity status (prepuberty or maturity; MATURITY) effects on endocrine milieu of the preovulatory ovarian follicle in gilts. HCG directly (blue solid arrows) affects STAR protein expression and steroid hormones (P_4_, A_4_, T) and PGE_2_ production. GnRH-A causes release of endogenous LH that after activation of LH/hCG receptor (LHCGR) affects (yellow solid arrows) HSD3B1, CYP11A1, FSH receptor (FSHR), PTGFS protein expression, estradiol (E_2_), prostaglandin F_2α_ (PGF_2α_) concentration in follicular fluid, matrix metalloproteinase (MMP-1) and its inhibitor (TIMP-1) proteins. MATURITY directly (red solid arrows) affects steroidogenic enzymes (STAR, CYP17A1) and LHCGR proteins, E_2_ concentration, transcription factors CREB1 and APF4 proteins, local regulators of steroidogenesis vimentin (VIM), transferrin (TF) and MMP-1, TIMP-1 proteins. The positive ( +) or negative (-) correlations between studied proteins and/or hormones are showed (double – faced dotted arrows).

In summary, the endocrine and molecular milieu of preovulatory follicles is governed by sexual maturity (prepuberty or maturity) as well as exogenous/endogenous gonadotropins (Fig. 8). Our data suggest that hCG increases progesterone and androgen production and weakens estrogen supply, while GnRH-A improves estrogen production and PGF_2α_ availability. Importantly, sexual maturity provides a stronger estrogenic environment. It directly affects factors involved in steroidogenesis (CYP17A1 and STAR), transcription factors (CREB1 and ATF4), LHCGR, local regulators of cell function (VIM and TF), and E_2_ concentration in the follicular fluid. While hCG affects STAR protein, P_4_, A_4_, T, and PGE_2_ concentration in follicular fluid, GnRH-A-induced LH affects HSD3B1, CYP11A1, FSHR, and PTGFS, as well as E_2_ and PGFM (PGF_2α_) concentration in the follicular fluid. These data support the concept of the different endocrine properties of LH and hCG, which comprise a potent progestational and androgenic role of hCG and pro-developmental and antiapoptotic action of LH^10^^,^^11^ in the control of porcine preovulatory follicles. These data contribute to a better understanding of how exogenous and native gonadotropins influence ovarian follicles, depending on sexual maturity. More research is still required to reveal the mechanisms governing the numerous relationships between exogenous/endogenous gonadotropins and sexual maturity during the development and function of preovulatory follicles.

## Supplementary Information

Supplementary Information.
