# Supplementary material for: Endocrine and molecular milieus of ovarian follicles are diversely affected by human chorionic gonadotropin and gonadotropin-releasing hormone in prepubertal and mature gilts
Source: Sci Rep. 2021 Jun 29;11:13465. doi: 10.1038/s41598-021-91434-6 (PMC8242046; doi:10.1038/s41598-021-91434-6)
Supplement: Supplementary file 1 — Supplementary Information. [file 41598_2021_91434_MOESM1_ESM.pdf]

Supplementary information

Endocrine and Molecular Milieus of Ovarian Follicles Are Diversely Affected by Human Chorionic Gonadotropin and Gonadotropin-Releasing Hormone in Prepubertal and Mature Gilts

Adam J. Ziecik, Jan Klos, Katarzyna Gromadzka-Hliwa, Mariola A. Dietrich, Mariola Slowinska, Pawel Likszo, Katarzyna Knapczyk-Stwora, Zdzislaw Gajewski and Monika M. Kaczmarek

Supplementary Tables:

Supplementary Table 1. List of antibodies used in the study.

Supplementary Table 2. Mixing and dying scheme of samples of follicular walls of hCG- or GnRH-A-treated prepubertal and mature gilts (n=5-6 per group) using Cy2, Cy3 and Cy5 CyDye DIGE Flour dyes.

Supplementary Table 3. List of genes and assays used in real-time PCR.

Supplementary Table 4. Ratio of hormone concentrations in the preovulatory follicular fluid of hCG- or GnRH-A-treated prepubertal or mature gilts.

Supplementary Table 5. Proteins abundance in follicular walls of hCG-treated prepubertal *vs* mature gilts.

Supplementary Table 6. Proteins abundance in follicular walls of GnRH-A-treated prepubertal *vs* mature gilts.

Supplementary Figures:

Supplementary Fig. 1. Number of ovarian follicles in three different size categories (<6, 6–8, and >8 mm in diameter) in prepubertal (A) and mature (B) gilts.

Supplementary Fig. 2. Hormones (hCG and GnRH; HORMONE) and sexual maturity (MAT) change the abundance of factors related to production of androgens and estrogens in ovarian follicles of prepubertal and mature gilts.

Supplementary Fig. 3A-E. Uncropped blots for protein expression in prepubertal and mature gilts challenged with hCG or GhRH-A.

Supplementary Table 1. List of antibodies used in the study.

Peptide/Protein Target	Name of Antibody	Catalog No., Name of Source	Species Raised in Monoclonal or Polyclonal	Dilution used
**Prostaglandin assays**				
PGE_2	Anti-Prostaglandin E_2 Antibody	P5164 Sigma-Aldrich	Rabbit, polyclonal	1:200
PGFM	Anti-PGFM serum	WS4468-7 donated by Dr William Silvia	Rabbit, polyclonal	1:10000
**Immunohistochemistry**				
TF	Anti-Transferrin Antibody	ab82411 Abcam	Rabbit, polyclonal	1:100
VIM	Anti-Vimentin Antibody	sc-6260 Santa Cruz Biotechnology	Mouse, monoclonal	1:50
Anti-Rabbit, secondary antibodies	Goat Anti-Rabbit IgG Antibody (H+L), Biotinylated	BA-1000-1.5 Vector Laboratories	Goat, polyclonal	1.300
Anti-Mouse, secondary antibodies	Horse Anti-Mouse IgG Antibody (H+L), Biotinylated	BA-2000-1.5 Vector Laboratories	Horse, polyclonal	1:300
**Western blot**				
ATF4	Anti-ATF4 Antibody	11815 Cell Signaling Technology	Rabbit, monoclonal	1:400
CREB1	Anti-CREB1 Antibody	sc186 Santa Cruz Biotechnology	Rabbit, polyclonal	1:100
CYP11A1	Anti- CYP11A1 Antibody	Ab75497 Abcam	Rabbit, polyclonal	1:750
CYP17A1	Anti- CYP17A1 Antibody	ab125022 Abcam	Rabbit, monoclonal	1:1000
CYP19A1	Anti-CYP19A1 Antibody	MCA2077S Bio-Rad	Mouse, monoclonal	1:250
FSHR	Anti-FSHR Antibody	bs 0895R Biossua	Rabbit, polyclonal	1:400
HSD3B1	Anti- HSD3B1 Antibody	ab55268 Abcam	Mouse, monoclonal	1:100
LHCGR	Anti- LHCGR Antibody	AD2716317 donated by Dr Marco Banoni	Mouse, monoclonal	1:600
MMP1	Anti-MMP1 Antibody	ab137332 Abcam	Rabbit, polyclonal	1:500
PTGFS	Anti- PTGFS Antibody	AV48180 Sigma	Rabbit, polyclonal	1:200
STAR	Anti-StAR Antibody	ab96637 Abcam	Rabbit, polyclonal	1:400
TF	Anti-Transferrin Antibody	ab82411 Abcam	Rabbit, polyclonal	1:10000
TIMP1	Anti- TIMP1 Antibody	sc21734 Santa Cruz Biotechnology	Mouse, monoclonal	1:400
WIM	Anti- WIM Antibody	sc-6260	Mouse, polyclonal	1:400

		Santa Cruz Biotechnology		
GAPDH	Anti- GAPDH Antibody	MA5-15738 Thermo-Fisher Scientific	Mouse, monoclonal	1:2000
Anti-rabbit, secondary antibodies	Immun-Star Goat Anti-Rabbit (GAR)-HRP Conjugate	1705046 Bio-Rad	Goat, polyclonal	1:20000
Anti-mouse, secondary antibodies	Immun-Star Goat Anti-Mouse (GAM)-HRP Conjugate	1705047 Bio-Rad	Goat, polyclonal	1:20000

Supplementary Table 2. Mixing and dying scheme of samples of follicular walls of hCG- or GnRH-A-treated prepubertal and mature gilts (n=5-6 per group) using Cy2, Cy3 and Cy5 CyDye DIGE Flour dyes.

Gel Number	Cy2	Cy3	Cy5
**1**	Pooled Standard	Mature gilts + hCG 1	Mature gilts + GnRH 4
**2**	Pooled Standard	Mature gilts + GnRH 1	Prepubertal gilts + hCG 4
**3**	Pooled Standard	Prepubertal gilts + hCG 1	Prepubertal gilts + GnRH 4
**4**	Pooled Standard	Prepubertal gilts + GnRH 1	Mature gilts + hCG 4
**5**	Pooled Standard	Mature gilts + hCG 2	Prepubertal gilts + hCG 5
**6**	Pooled Standard	Mature gilts + GnRH 2	Prepubertal gilts + GnRH 5
**7**	Pooled Standard	Prepubertal gilts + hCG 2	Mature gilts + hCG 5
**8**	Pooled Standard	Prepubertal gilts + GnRH 2	Mature gilts + GnRH 5
**9**	Pooled Standard	Mature gilts + hCG 3	Mature gilts + GnRH 6*
**10**	Pooled Standard	Mature gilts + GnRH 3	Prepubertal gilts + hCG 6
**11**	Pooled Standard	Prepubertal gilts + hCG 3	Prepubertal gilts + GnRH 6
**12**	Pooled Standard	Prepubertal gilts + GnRH 3	Mature gilts + hCG 6*

*Technical replicate

Supplementary Table 3. List of genes and assays used in real-time PCR.

Genes symbol	Gene name	TaqMan Assays ID
STAR	Steroidogenic Acute Regulatory Protein	Ss03381250_u1
HSD3B1	Hydroxy-delta-5-steroid Dehydrogenase, 3 Beta- and Steroid Delta-isomerase 1	Ss03391752_m1
CYP17A1	Cytochrome P450 Family 17 Subfamily A Member 1	Ss03394945_m1
CYP19A1	Cytochrome P450 Family 19 Subfamily A Member 1	Ss03384876_u1
PTGS2	Prostaglandin-Endoperoxide Synthase 2	Ss03394694_m1
PTGES	Prostaglandin E Synthase	Ss03392129_m1
PTGFS	Prostaglandin F Synthase	NM_001038626
MMP1	Matrix Metallopeptidase 1	Ss04245662_m1
TIMP1	Tissue Inhibitor Of Metalloproteinases 1	Ss03381944_u1
CREB1	CAMP Responsive Element Binding Protein 1	Ss03386122_u1
ATF4	Activating Transcription Factor 4	Ss03390207_g1
LHCGR	Luteinizing Hormone/Choriogonadotropin Receptor	Ss03384991_u1
FSHR	Follicle Stimulating Hormone Receptor	Ss03384581_u1
*ACTB**	Beta-Actin	Ss03376081_u1
*GAPDH**	Glyceraldehyde 3-phosphate Dehydrogenase	Ss03375435_u1
*HPRT1**	Hypoxanthine–guanine Phosphoribosyltransferase	Ss03388274_m1

*Reference genes

Supplementary Table 4. Ratio of hormone concentrations in the preovulatory follicular fluid of hCG- or GnRH-A-treated prepubertal or mature gilts.

**MATURITY STATUS**	**PREPUBERTAL**		**MATURE**		**MAIN EFFECT**
**HORMONES**	**hCG**	**GnRH-A**	**hCG**	**GnRH-A**	
**P_4/E_2**	34.4 ± 6.0^a	11.9 ± 3.7^b	19.7 ± 3.7	7.2 ± 2.0	MAT P = 0.045<br>HORMONE P = 0.0014
**T/E_2**	38.2 ± 5.3^a	8.3 ± 2.9^b	28.5 ± 5.2^A	6.0 ± 0.2^B	HORMONE P = 0.0002
**A_4/E_2**	17.2 ± 5.2^a	1.7 ± 0.6^b	10.2 ± 3.7	1.2 ± 0.2	HORMONE P = 0.0006
**P_4/T**	0.9 ± 0.1	1.2 ± 0.5	0.7 ± 0.1	1.2 ± 0.3	
**P_4/A_4**	2.2 ± 0.2	6.4 ± 2.1	1.5 ± 0.2	7.4 ± 1.0	HORMONE P = 0.006
**T/A_4**	2.8 ± 0.4	4.8 ± 1.0	2.4 ± 0.3	5.4 ± 0.7	HORMONE P = 0.02

Data are expressed as mean ± SEM. Means with different superscripts differ significantly (small letters - prepubertal gilts, capital letters - mature gilts; $P < 0.05$).

Supplementary Table 5. Proteins abundance in follicular walls of hCG-treated prepubertal *vs.* mature gilts.

Spot no.	Identified protein	Protein symbol	P value	Fold change	NCBI accession number	MW (kDa/pI)
484	albumin, partial [*Sus scrofa*]	ALB	0.00052	2.13	113576	71362/5.92
840	albumin, partial [*Sus scrofa*]	ALB	0.025	2.1	113576	71362/5.92
337	transferrin, partial [*Sus scrofa*]	TF	0.0023	1.98	115394517	78954/6.73
323	transferrin, partial [*Sus scrofa*]	TF	0.0011	1.94	115394517	78954/6.73
662	serpin A3-8 [*Sus scrofa*]	SERPINA3-8	0.0077	1.92	112874	47168/6.68
458	albumin, partial [*Sus scrofa*]	ALB	0.00071	1.79	113576	71362/5.92
535	albumin, partial [*Sus scrofa*]	ALB	0.0029	1.79	113576	71362/5.92
345	transferrin, partial [*Sus scrofa*]	TF	0.0045	1.69	115394517	78954/6.73
429	albumin, partial [*Sus scrofa*]	ALB	0.0027	1.68	113576	71362/5.92
422	albumin, partial [*Sus scrofa*]	ALB	0.0054	1.66	113576	71362/5.92
339	albumin, partial [*Sus scrofa*]	ALB	0.0073	1.64	113576	71362/5.92
120	ceruloplasmin precursor [*Sus scrofa*]	CP	0.030	1.44	1519314162	122636/5.72
212	complement factor B [*Sus scrofa*]	CFB	0.010	1.38	297569	87239/7.45
529	albumin, partial [*Sus scrofa*]	ALB	0.022	1.38	113576	71362/5.92
465	albumin, partial [*Sus scrofa*]	ALB	0.0062	1.37	113576	71362/5.92
161	plasminogen precursor [*Sus scrofa*]	PLG	0.040	1.34	130316	93293/7.00
272	prelamin-A/C [*Sus scrofa*]	LMNA	0.015	1.29	125962	74416/6.73
608	IgG heavy chain precursor [*Sus scrofa*]	IGHG	0.024	1.27	32011	51583/6.66
343	transferrin, partial [*Sus scrofa*]	TF	0.035	1.23	115394517	78954/6.73
268	plasma gelsolin precursor, partial [*Sus scrofa*]	GSN	0.015	1.22	121116	85065/5.93
456	albumin, partial [*Sus scrofa*]	ALB	0.0050	1.22	113576	71362/5.92
1512	stathmin [*Sus scrofa*]	STMN1	0.049	1.2	57870	17292/5.76
271	histidine-rich glycoprotein precursor [*Sus scrofa*]	HRG	0.030	1.17	4504489	62255/7.19

271 a	prelamin-A/C [*Sus scrofa*]	LMNA	0.030	1.17	125962	74416/6.73
430	78 kDa glucose-regulated protein isoform X2 [*Sus scrofa*]	HSPA5	0.026	1.16	14916999	72338/5.06
1241	peptidyl-prolyl cis-trans isomerase FKBP3 [*Sus scrofa*]	FKBP3	0.028	-1.15	4503727	25219/9.29
1271	PREDICTED: phosphoglycerate mutase 1-like [*Sus scrofa*]	PGAM1	0.0042	-1.16	130348	28900/6.67
914	serpin H1 precursor [*Sus scrofa*]	SERPINH1	0.050	-1.18	333360851	46648/8.91
1244	peptidyl-prolyl cis-trans isomerase FKBP3 [*Sus scrofa*]	FKBP3	0.013	-1.18	194097323	25219/9.29
607	fibrinogen beta chain precursor [*Sus scrofa*]	FGB	0.040	-1.19	399492	57107/8.11
288	lipoma-preferred partner [*Sus scrofa*]	LPP	0.048	-1.22	1915602459	67033/7.45
905	cytoskeletal beta actin, partial [*Sus scrofa*]	ACTB	0.046	-1.22	296434507	45162/5.55
911	cytoskeletal beta actin, partial [*Sus scrofa*]	ACTB	0.046	-1.22	296434507	45162/5.55
616	protein disulfide-isomerase A3 precursor [*Sus scrofa*]	PDIA3	0.020	-1.23	21361657	57279/5.93
1522	cytochrome b5 [*Sus scrofa*]	CYB5A	0.024	-1.27	117809	15301/5.04
1117	dehydrogenase, glyceraldehydephosphate [*Sus scrofa*]	GAPDH	0.027	-1.28	224880	35915/6.63
283	vimentin isoform X1 [*Sus scrofa*]	VIM	0.021	-1.33	62414289	53692/5.06
294	vimentin isoform X1 [*Sus scrofa*]	VIM	0.022	-1.35	62414289	53692/5.06
1059	tropomyosin beta chain [*Sus scrofa*]	TPM2	0.032	-1.36	669033277	33383/4.62
237	vimentin isoform X2 [*Sus scrofa*]	VIM	0.0040	-1.38	62414289	49248/5.09
460	heat shock cognate 71 kDa protein [*Sus scrofa*]	HSPA8	0.021	-1.38	5729877	71050/5.37
805	cholesterol side-chain cleavage enzyme, mitochondrial precursor [*Sus scrofa*]	CYP11A1	0.032	-1.4	153218646	60447/9.06
807	cholesterol side-chain cleavage enzyme, mitochondrial precursor [*Sus scrofa*]	CYP11A1	0.027	-1.4	153218646	60447/9.06

1641	hemoglobin subunit alpha [*Sus scrofa*]	HBA1	0.039	-1.41	57013850	15218/8.74
543	T-complex protein 1 subunit epsilon isoform X1 [*Sus scrofa*]	CCT5	0.010	-1.45	24307939	62687/5.73
604	protein disulfide-isomerase A3 precursor [*Sus scrofa*]	PDIA3	0.00096	-1.54	21361657	57279/5.93
342	alpha-1B-glycoprotein [*Sus scrofa*]	A1BG	0.012	-1.55	119592981	54933/5.99
1110	annexin A2 isoform X1 [*Sus scrofa*]	ANXA2	0.0088	-1.55	50845386	38840/6.92
1644	hemoglobin subunit alpha [*Sus scrofa*]	HBA1	0.0033	-1.57	57013850	15218/8.74
803	cholesterol side-chain cleavage enzyme, mitochondrial precursor [*Sus scrofa*]	CYP11A1	0.018	-1.66	153218646	60447/9.06
174	desmin, partial [*Sus scrofa*]	DES	0.046	-1.71	6686280	52684/5.21
663	serpin A3-8 [*Sus scrofa*]	SERPINA3-8	0.045	-7.51	112874	46782/5.80

Supplementary Table 6. Proteins abundance in follicular walls of GnRH-A-treated prepubertal *vs.* mature gilts.

Spot no.	Identified protein	Protein symbol	P value	Fold change	NCBI accesion number	MW (kDa/pI)
572a	fibrinogen beta chain precursor [*Sus scrofa*]	FGB	0.013	1.46	399492	57107/8.11
572b	PREDICTED: catalase [*Equus asinus*]	CAT	0.013	1.46	4557014	55611/6.86
572c	stress-induced-phosphoprotein 1 [*Sus scrofa*]	STIP1	0.013	1.46	12804257	63052/6.36
1260	enoyl-CoA hydratase. mitochondrial [*Sus scrofa*]	ECHS1	0.025	1.33	194097323	31558/8.81
1549	peptidyl-prolyl cis-trans isomerase *A* [*Sus scrofa*]	PPIA	0.0011	1.27	10863927	18086/8.34
294	vimentin isoform X1 [*Sus scrofa*]	VIM	0.025	1.23	62414289	53692/5.06
1623	galectin-1 partial [*Sus scrofa*]	LGALS1	0.018	1.19	4504981	14932/5.07
1090	heterogeneous nuclear ribonucleoprotein A2/B1 [*Sus scrofa*]	HNRNPAB1	0.011	1.16	133257	36041/8.76
1055	PREDICTED: heterogeneous nuclear ribonucleoproteins A2/B1 isoform 1 [*Sus scrofa*]	HNRNPAB1	0.027	1.15	133257	37464/8.97
983	PREDICTED: heterogeneous nuclear ribonucleoprotein D0 isoform X4 [*Nannospalax galili*]	HNRNPD	0.044	1.15	51477708	30373/8.53
721	thioredoxin domain-containing protein 5 [*Sus scrofa*]	TXNDC5	0.019	-1.17	29839560	48701/5.94
1135	LIM and SH3 domain protein 1 isoform X1 [*Sus scrofa*]	LASP1	0.016	-1.18	5453710	30068/6.61
1080	Chain A. X-Ray Structure Of Full-Length Annexin 1 [*Sus scrofa*]	ANCA1	0.0087	-1.24	4502101	39005/6.37
696	fibrinogen gamma chain isoform X1 [*Sus scrofa*]	FGG	0.030	-1.25	20178280	51348/5.89
1203	voltage-dependent anion-selective channel protein 1 [*Sus scrofa*]	VDAC1	0.033	-1.26	4507879	30822/8.62
161	plasminogen precursor [*Sus scrofa*]	PLG	0.043	-1.29	130316	93293/7.00
434	alpha-1B-glycoprotein [*Sus scrofa*]	AHSG	0.017	-1.3	178284	54933/5.99

162	plasminogen precursor [*Sus scrofa*]	PLG	0.043	-1.35	130316	93293/7.00
697	fibrinogen gamma chain isoform X1 [*Sus scrofa*]	FGG	0.034	-1.36	20178280	51348/5.89
177	PREDICTED: major vault protein isoform 1 [*Sus scrofa*]	MVP	0.0096	-1.42	15990478	99935/5.57
152	inter-alpha-trypsin inhibitor heavy chain H4 isoform X10 [*Sus scrofa*]	ITIH4	0.044	-1.45	229463048	107348/6.10
120	ceruloplasmin precursor [*Sus scrofa*]	CP	0.038	-1.51	1519314162	122636/5.72
119	ceruloplasmin precursor [*Sus scrofa*]	CP	0.044	-1.55	1519314162	122636/5.72
558	fibrinogen beta chain precursor [*Sus scrofa*]	FGB	0.020	-1.58	399492	57107/8.11
605	vitamin D-binding protein [*Sus scrofa*]	GC	0.017	-1.77	1476413323	54724/5.38
117	ceruloplasmin precursor [*Sus scrofa*]	CP	0.0047	-1.83	1519314162	122636/5.72
170	inter-alpha-trypsin inhibitor heavy chain H4 isoform X11 [*Sus scrofa*]	ITIH4	0.0040	-1.83	229463048	100792/6.35
165	inter-alpha-trypsin inhibitor heavy chain H4 isoform X11 [*Sus scrofa*]	ITIH4	0.0030	-1.91	229463048	100792/6.35
169	inter-alpha-trypsin inhibitor heavy chain H4 isoform X2 [*Sus scrofa*]	ITIH4	0.019	-1.91	229463048	110904/6.30
159	Chain A. Crystal Structure Of Bovine Serum Albumin [*Bos taurus*]	ALB	0.022	-1.97	113576	68416/5.60
520	LOW QUALITY PROTEIN: serpin A3-5 [*Sus scrofa*]	SERPINA-5	0.0085	-2.39	112874	47593/5.81
545	LOW QUALITY PROTEIN: serpin A3-8 [*Sus scrofa*]	SERPINA-8	0.012	-3.28	112874	47455/7.72

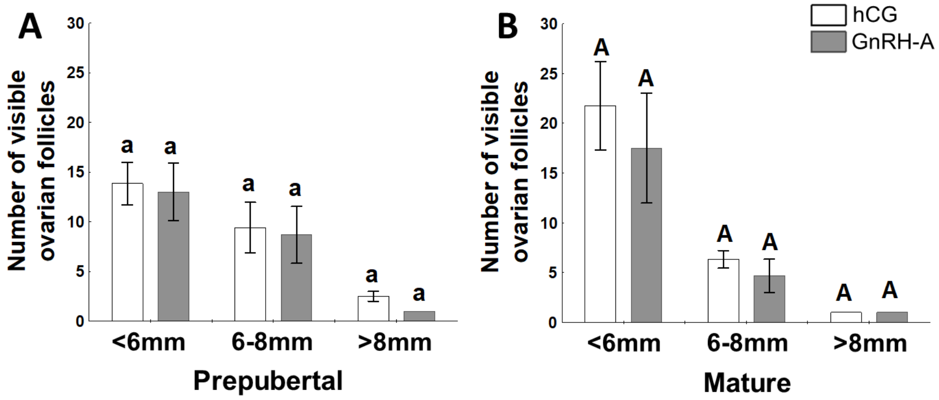

Supplementary Fig. 1. Number of ovarian follicles in three different size categories (<6, 6–8, and >8 mm in diameter) in prepubertal (A) and mature (B) gilts. Data were analyzed using two-way ANOVA (main effects: HORMONE, MAT; not significant) and are presented as mean ± SEM (n = 4–6 per group).

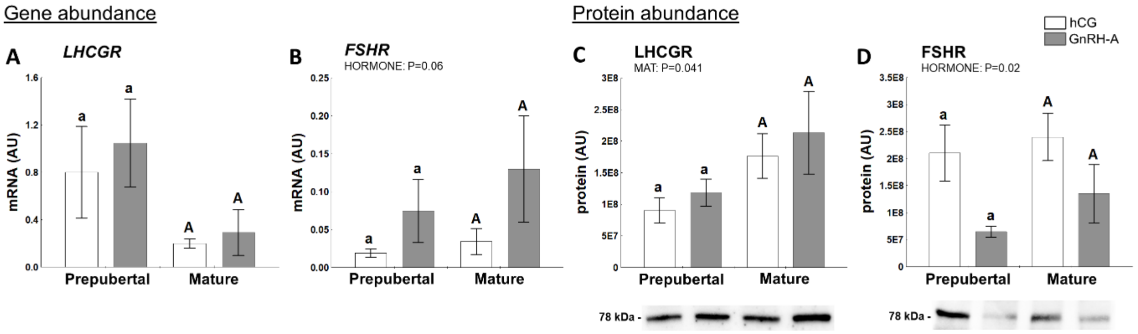

Supplementary Fig. 2. Hormones (hCG and GnRH; HORMONE) and sexual maturity (MAT) change the abundance of factors related to production of androgens and estrogens in ovarian follicles of prepubertal and mature gilts. The expression of LHCGR (A, C), and FSHR (B, D) in prepubertal and mature gilts was evaluated. Gene expression was normalized to the geometric mean of ACTB and GAPDH (AU), identified as the best reference genes by NormFinder algorithm. Protein levels were normalized to total protein levels (AU) using TGX Stain-Free gel technology (C, D). Uncropped blots are presented in Supplementary Fig. 3E online. Data were analyzed using two-way ANOVA with Sidak multiple comparison (mRNA) or Tukey post-hoc (protein) tests (main effects: HORMONE, MAT) and are presented as mean ± SEM (n = 4–6 per group). Means with different superscripts differ significantly (small letters - prepubertal gilts, capital letters - mature gilts; P < 0.05). AU – arbitrary units.

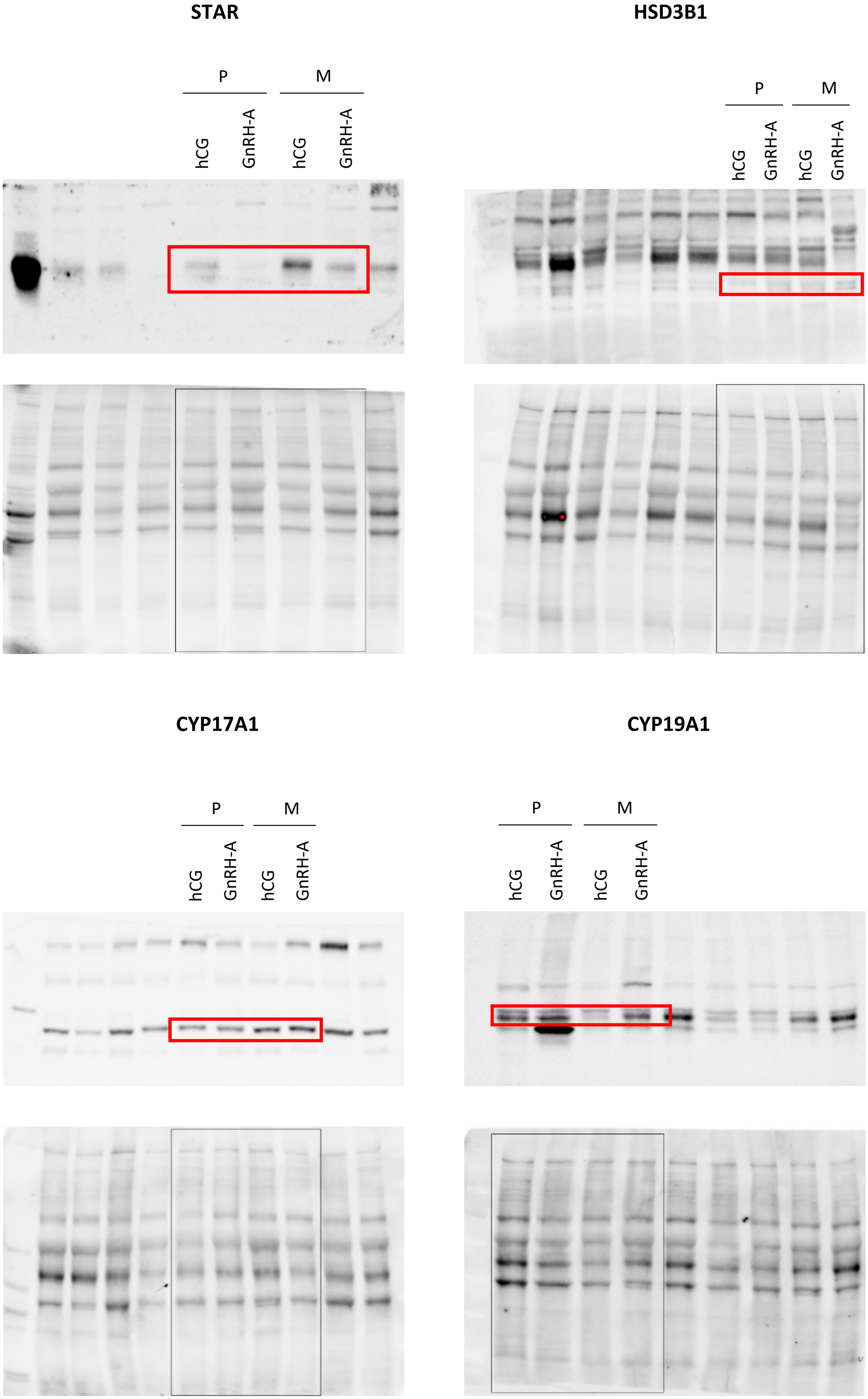

Supplementary Fig. 3A. Uncropped blots for STAR, HSD3B1, CYP17A1 and CYP19A1 protein expression in prepubertal and mature gilts challenged with hCG or GhRH-A. In each upper panel, full blot showing STAR, HSD3B1, CYP17A1 and CYP19A1 protein expression are showed. Lower panels represent equivalent TGX Stain-Free gel showing total protein. Red boxes indicate areas presented in Figure 2. P - prepubertal, M - mature

PTGFS

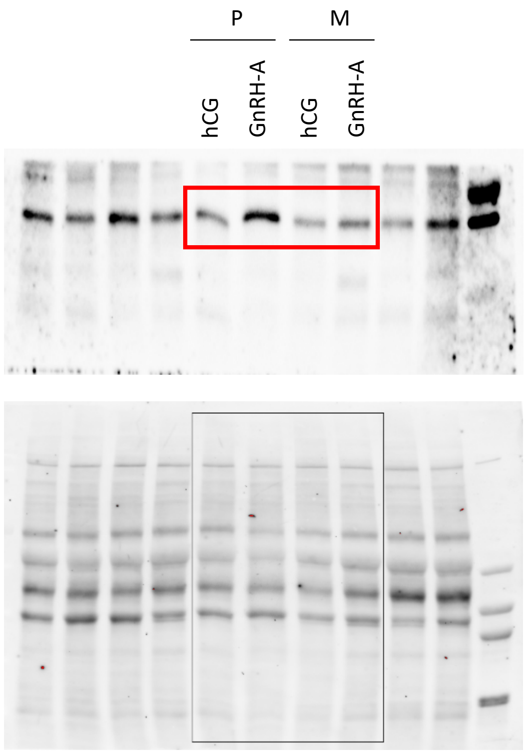

Supplementary Fig. 3B. Uncropped blots for PGFS protein expression in prepubertal and mature gilts challenged with hCG or GhRH-A. In each upper panel, full blot showing PTGFS protein expression are showed. Lower panel represent equivalent TGX Stain-Free gel showing total protein. Red boxes indicate areas presented in Figure 3. P - prepubertal, M – mature

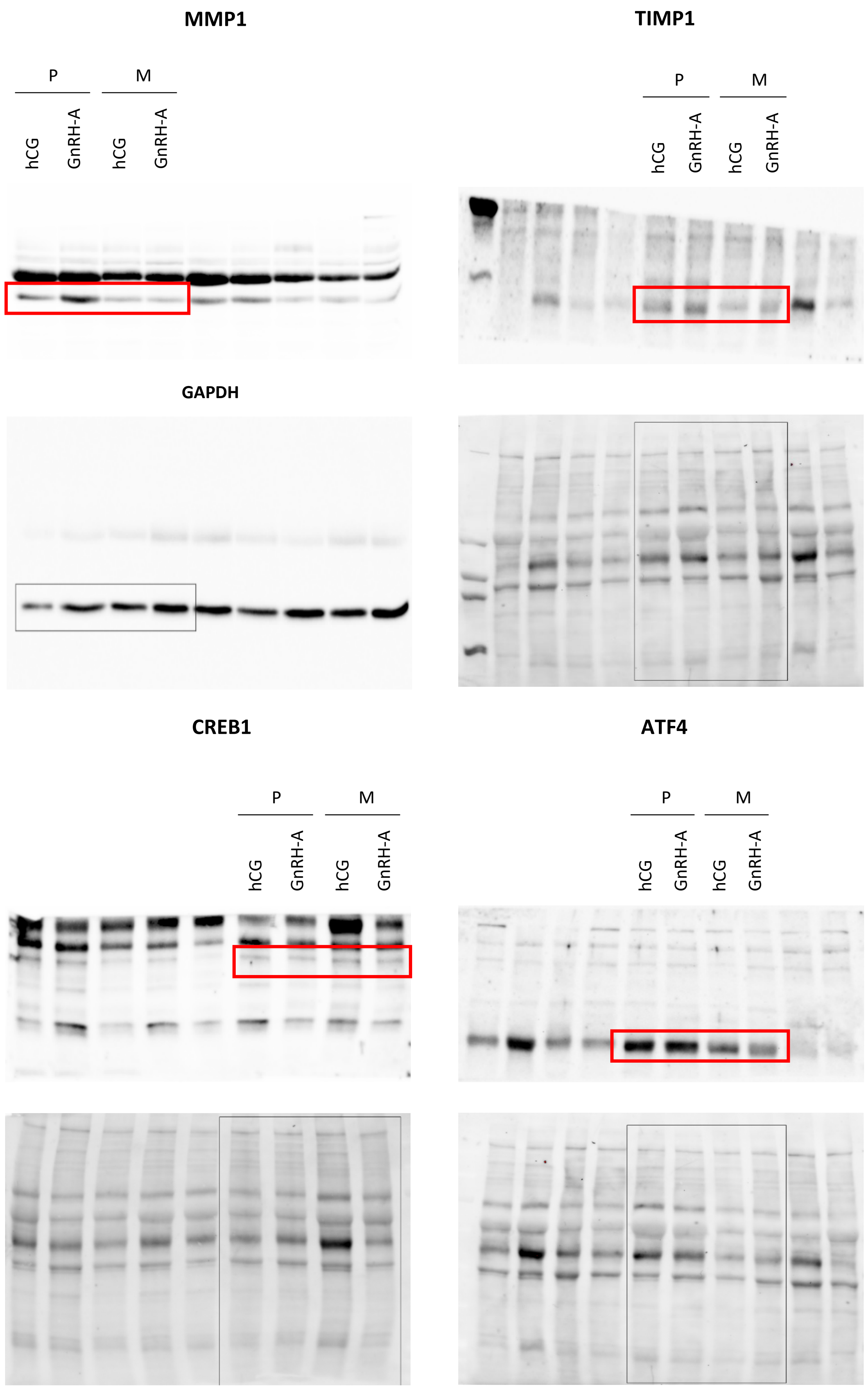

Supplementary Fig. 3C. Uncropped blots for MMP1, TIMP1, CREB1 and ATF4 protein expression in prepubertal and mature gilts challenged with hCG or GhRH-A. In each upper panel, full blot showing MMP1, TIMP1, CREB1 and ATF4 protein expression are showed. Lower panels represent equivalent TGX Stain-Free gel showing total protein, MMP1 (GAPDH). Red boxes indicate areas presented in Figure 4 and 5. P - prepubertal, M - mature

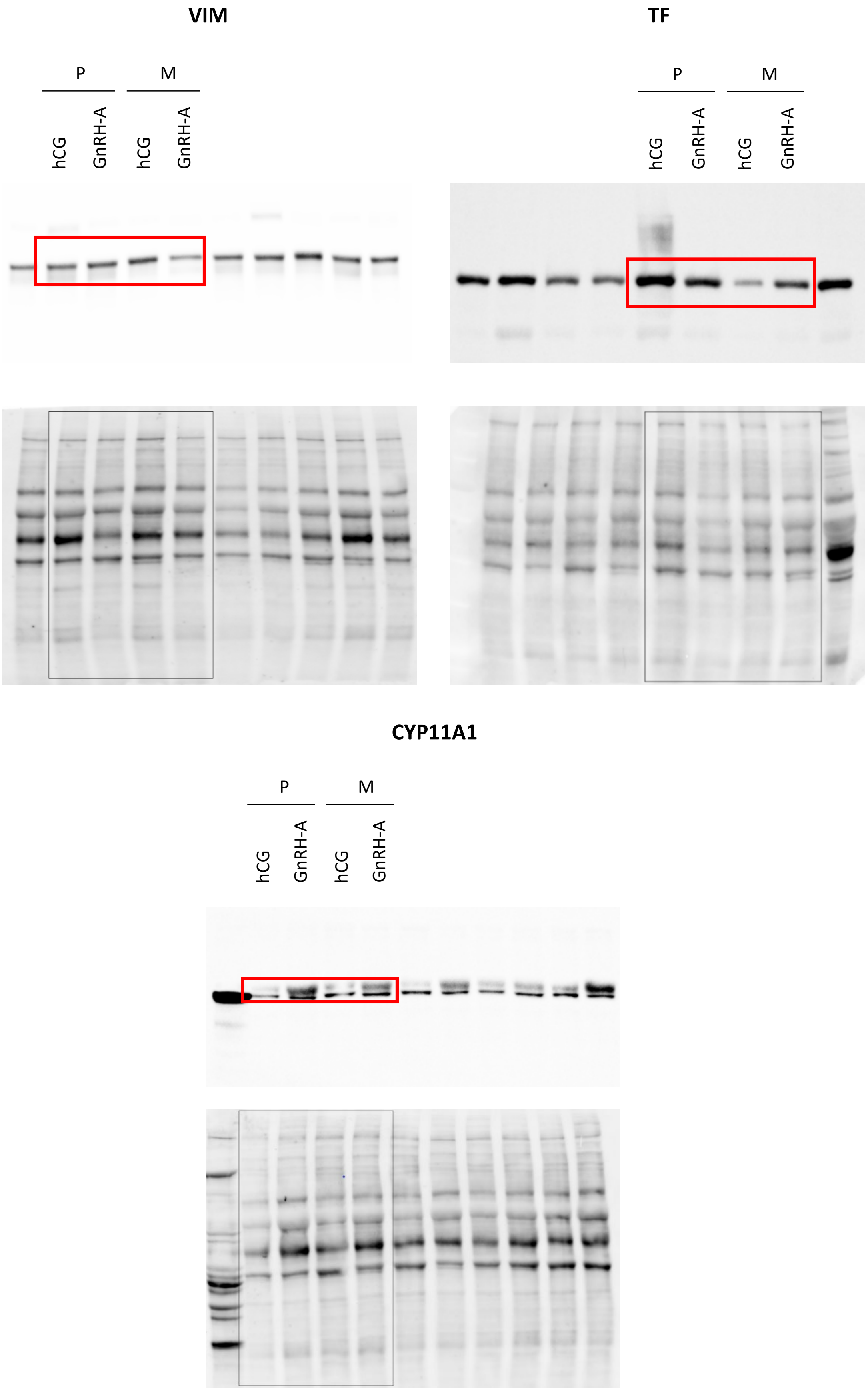

Supplementary Fig. 3D. Uncropped blots for VIM, TF and CYP11A1 protein expression in prepubertal and mature gilts challenged with hCG or GhRH-A. In each upper panel, full blots for VIM, TF and CYP11A1 protein expression are showed. Lower panels represent equivalent TGX Stain-Free gel showing total protein. Red boxes indicate areas presented in Figure 7. P - prepubertal, M - mature

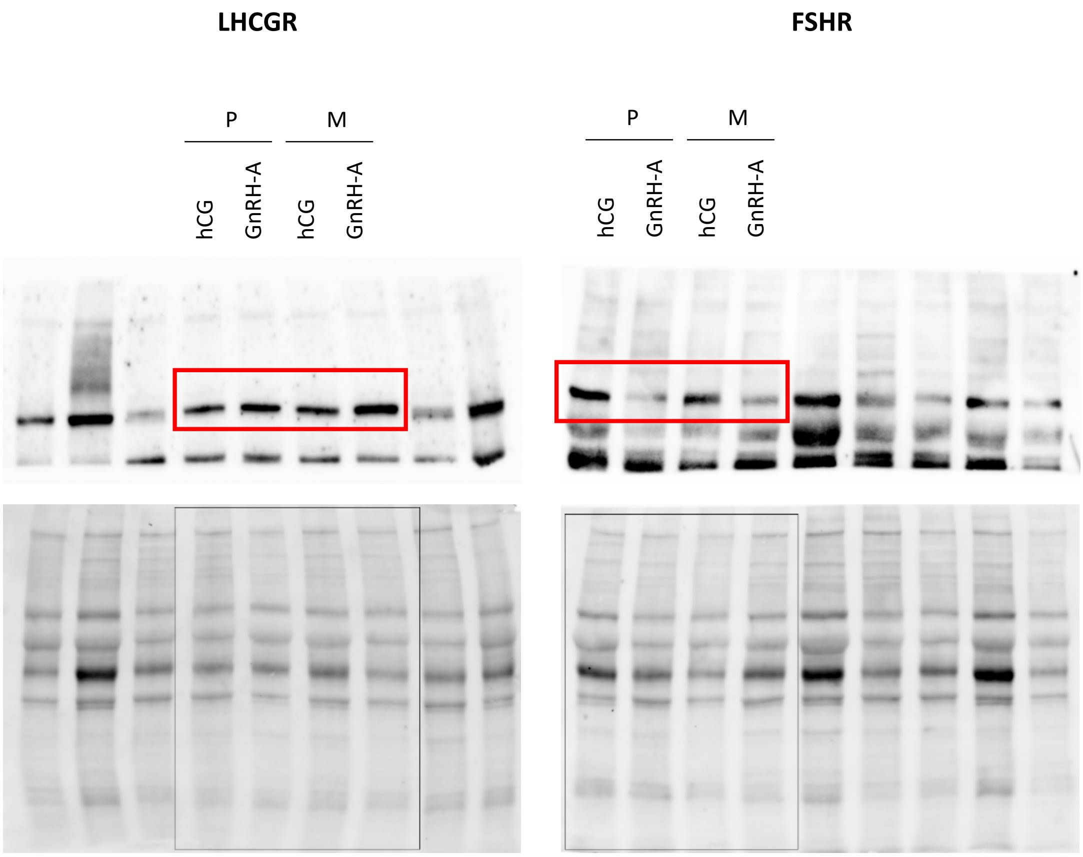

Supplementary Fig. 3E. Uncropped blots for LHCGR and FSHR protein expression in prepubertal and mature gilts challenged withhCG or GhRH-A. In each upper panel, full blot showing LHCGR and FSHR protein expression are showed. Lower panels represent equivalent TGX Stain-Free gel showing total protein. Red boxes indicate areas presented in Supplementary Figure 2. P - prepubertal, M - mature
